# Prediabetes in Colombia: Expert Consensus

**DOI:** 10.25100/cm.v43i4.3662

**Published:** 2017-12-30

**Authors:** Patricio López-Jaramillo, Carlos Calderón, Jorge Castillo, Iván Darío Escobar, Enrique Melgarejo, Gustavo Adolfo Parra

**Affiliations:** 1 Director de Investigación, Desarrollo e Innovación Tecnológica, Clínica de Síndrome Metabólico, Prediabetes y Diabetes, Bucaramanga, Colombia; 2 Fundación Oftalmológica de Santander (FOSCAL), Bucaramanga, Colombia; 3 Presidente de la Federación Diabetológica Colombiana (FDC), Bogotá, Colombia; 4 Director de la Fundación Santandereana de Diabetes (FUSANDE), Bucaramanga, Colombia.; 5 Presidente de la Sociedad Colombiana de Sarcopenia (SCS), Bogotá, Colombia.; 6 Vicepresidente de la Federación Diabetológica Colombiana (FDC), Bogota, Colombia.; 7 Ex Presidente de la Fundación Colombiana de Obesidad (FUNCOBES), Bogotá, Colombia.; 8 Delegado de la Asociación Colombiana de Endocrinología (ACE), Bogota, Colombia; 9 Presidente de la Sociedad Colombiana de Cardiología y Cirugía Cardiovascular (SCC), Bogotá, Colombia.; 10 Presidente Honorario del Colegio Panamericano del Endotelio (CPE), Bogotá, Colombia.; 11 Presidente de la Asociación Colombiana de Medicina Interna (ACMI), Bogotá, Colombia.; 12 Universidad Autonoma de Bucaramanga (UNAB), Bucaramanga, Colombia

**Keywords:** Prediabetes, diabetes mellitus type 2, cardiovascular diseases, Colombia, Prediabetes, Diabetes Mellitus tipo 2, enfermedades cardiovasculares, Colombia

## Abstract

The prevalence of Prediabetes in Colombia is high, and despite being recognized and categorized in the main Medical Guidelines and included in the International Classification of Diseases in Colombia, knowledge and awareness of it is limited amongst healthcare professionals and in the community. Our expert group recommends that educational programs emphasize a global approach to risk which includes a recognition of the importance of prediabetes and its evaluation along with and other risk factors such as a family history of DM2, overweight and obesity, dislipidemia and hypertension. Studies conducted in Colombia demonstrate the value of the FINDRIS questionnaire as a tool to identify subjects at risk of prediabetes and DM2, and we recommend that it should be systematic applied throughout the country as part of government policy.

Prediabetes progresses to DM2 at an annual rate of 10%, but it has also been shown that prediabetes is an independent risk factor for cardiovascular outcomes. On this basis, the Committee recommends that once prediabetes is detected and diagnosed, immediate management of the disease begins through lifestyle changes, with follow up assessments performed at 3 and 6 months. If the patient does not respond with a weight loss of at least 5% and if the HbA1C values ​​are not normalized, pharmacological management should be initiated with a metformin dose of 500 mg / day, increasing up to 1,500 - 1,700 mg / day, according to tolerance.

## Resumen

La prevalencia de Prediabetes en Colombia es alta y a pesar estar reconocida y categorizada en las principales Guías Médicas y estar incluida en la Clasificación Internacional de Enfermedades vigente en Colombia, el conocimiento que de ella tiene el equipo de salud y la comunidad es limitada. El grupo de expertos recomienda que en los programas educativos se insista en un enfoque global del riesgo incluyendo la importancia de conocer y evaluar la prediabetes y otros factores de riesgo como antecedentes familiares de DM2, sobrepeso y obesidad, dislipidemia e hipertensión.

Estudios realizados en Colombia demuestran la utilidad de la encuesta FINDRIS para identificar sujetos en riesgo de prediabetes y DM2 por lo que se recomienda que la aplicación del FINDRIS debe ser una política gubernamental y aplicada en todo el país.

La Prediabetes progresa hacia DM2 a una tasa anual del 10% y se ha demostrado que independientemente de esto la Prediabetes es un factor de riesgo para desenlaces cardiovasculares por lo que el Comité recomienda que una vez detectada y diagnosticada, se inicie el manejo inmediato a través de cambios en el estilo de vida y realizar valoración a los 3 y 6 meses. Si el paciente no responde con una pérdida de peso de al menos el 5% y si no se normalizan los valores de HbA1C, iniciar manejo farmacológico con una dosis de 500 mg/día de metformina, escalando hasta 1,500 - 1,700 mg/día, según tolerancia.

## Introduction 

In August 2017, the Colombian Diabetological Federation convened a meeting with representatives of the Colombian Association of Endocrinology (ACE), the Colombian Association of Internal Medicine (ACMI), the Colombian Society of Cardiology (SCC), the Colombian Obesity Foundation (FUNCOBES), the Colombian Society of Sarcopenia (SCS) and the Latin American Society of Hypertension (LASH) to review current concepts, worldwide and particularly Colombian epidemiology, and the implications in the pathophysiology of cardiovascular diseases (CVD) of the glucose alterations that precede diabetes mellitus type 2 (DM2), known as prediabetes. In addition, to evaluate international and national guidelines for its definition, risk factors, diagnosis, pharmacological and non-pharmacological treatment and short and long term monitoring recommendations. This document will contribute to improving the knowledge of general practitioners and specialists about this blood glucose alteration associated with an increased risk for DM2 and CVD ^1^ ^,^ ^2^.

## Definition and diagnostic criteria for prediabetes

The term prediabetes refers to a metabolic state intermediate between normal glucose homeostasis and DM2. It is diagnosed via assessment of venous blood glucose levels, according the following criteria: impaired fasting blood glucose (IFG) values between 100 and 125 mg/dL after at least 8 hours of fasting, and/or glucose intolerance (ITG) when glycaemia values are between 140 and 199 mg/dL ​​ two hours post oral administration of a 75 g glucose load (OGTT), and/or if the values ​​of glycosylated haemoglobin (HbA1c) are between 5.7% and 6.4% ^1^. Other terms used to refer to prediabetes are: a category glycaemia associated with an increased risk of DM2, and intermediate hyperglycaemia, a term proposed by the World Health Organization (WHO) ^3^.

## Epidemiology of prediabetes

According to the International Diabetes Federation (IDF), the prevalence of prediabetes varies between 6% and 14% worldwide, and in Colombia the age adjusted estimate amongst 20-79-year-old adults is 8 to 10% ^4^.

The 2010 National Survey of the Nutritional Situation in Colombia (ENSIN) ^5^ reported a 5% incidence of prediabetes. However, glucose was measured in capillary blood, a method that underestimates blood glucose levels and may have led to an underestimation of prediabetes prevalence ^6^. This possibility is also supported by the fact that a high prevalence of overweight and obesity, conditions closely associated with prediabetes, was also reported in the survey; 50% of adults over 35 years of age were overweight or obese. In over 18 year olds, the prevalence of overweight and abdominal obesity increased substantially between the 2005 ^7^ and 2010 ^5^ ENSIN surveys (Table 1). The 2010 ENSIN reported a prevalence of overweight of 17% among children and adolescents aged 10 to 17 ^5^. The Prospective Urban and Rural Epidemiology (PURE) study - Colombia, that was implemented in 11 departments (states) across the country and included 7,500 adults aged 35 to 70, reported a prevalence of prediabetes of 11.9% ^8^ while a study conducted in the coastal city of Barranquilla ^9^ in adults of both sexes found a prevalence of isolated glucose intolerance of 8%, altered fasting glycaemia of 11% and prediabetes prevalence in 439 adults with a first myocardial infarction was 29.6 % ^10^. This data demonstrates the importance of prediabetes both due to its high prevalence among the Colombian adult population, as well as its relationship with cardiovascular outcomes ^11^.

**Table 1:** Prevalence of abdominal obesity in Colombia. ENSIN 2005 - 2010.

Age (years)	Abdominal obesity		Abdominal obesity	
	Men ≥90 cm		Women ≥80 cm	
	2005 (%)	2010 (%)	2005 (%)	2010 (%)
18 to 22	6.6	8.9	18.9	26.7
23 to 27	16.3	22.4	31.5	40.3
28 to 32	28.7	44.0	34.7	54.6
33 to 37	36.1	42.7	52.5	635
38 to 42	44.2	59.0	51.6	71.1
43 to 47	46.8	54.0	65.9	75.1
48 to 52	54.1	73.0	58.8	81.3
53 to 57	56.2	59.4	75.8	85.2
58 to 64	55.2	60.7	77.4	84.9
Total	33.8	39.8	51.4	62.0

Source: ENSIN 2005-2010

ENSIN: Spanish acronyms of National Survey of Nutritional Situation in Colombia 2010

## Knowledge and sub-diagnosis of prediabetes

Although prediabetes is recognized worldwide and accepted by the major national and international DM related guidelines, there is still a clear lack of knowledge and awareness among physicians and other health professionals about the condition, and as a consequence a lack of evaluation of this important metabolic state ^12^. A recent study among primary care physicians in the United States of America (USA), found that only 11% of respondents correctly answered all of a series of questions about prediabetes risk factors ^13^. In Colombia ^14^ the same survey was applied in 429 physicians who attended two internal medicine and diabetes academic events, finding that 9.5% were able to identify the twelve risk factors proposed in the survey.

The code for prediabetes exists in the International Classification of Diseases (ICD-10) applied in Colombia, and is classified as symptoms, signs and abnormal clinical and laboratory findings with the R730 Code, corresponding to abnormalities of the glucose tolerance test ^15^ (Table 2). This code has not been sufficiently disseminated among Colombian physicians, which may be contributing to an under-diagnosis of prediabetes, a situation that we suspect despite available data in Colombia regarding the diagnosis of prediabetes being scarce, brief and of poor methodological quality. The study, characterization of patients with Prediabetes in the first level of institutional care aimed to evaluate whether there was diagnosis of prediabetes in patients affiliated with a private health insurer known in Colombia as EPS (Health Promoting Entity) and found that despite the existence of factors associated with prediabetes, the diagnosis was not established nor was any specific therapeutic management proposed ^16^.

**Table 2:** ICD 10 code applied to pre-diabetes ^14^.

Chapter	Chapter title	ICD-10 three character code	Three character code description	ICD-10 Four character code	Four character code description	Inclusion
18	Abnormal clinical and laboratory symptoms, signs and ﬁndings not classiﬁed elsewhere	R73	Increased blood glucose level			Excludes: - Diabetes mellitus (E10-E14) - Pregnancy, childbirth and the puerperium (O24.-) - Post procedural hypoinsulinaemia (E89.1) - Neonatal disorders(P70.0-P70.2)
18	Abnormal clinical and laboratory symptoms, signs and ﬁndings not classiﬁed elsewhere	R73	Increased blood glucose level	R730	Glucose intolerance testing abnormalities	DIABETES: -Lactante Química
						PREDIABETES:-Impaired glucose tolerance
18		R73		R739		

## Progression of prediabetes to DM2 and impact on cardiovascular events

DM2 is a progressive disease that begins with a long asymptomatic phase in individuals with various risk factors ^17^ which have been identified in population studies such as the PURE-Global study. Older age, male sex, urban residence, low educational level, low physical activity, family history of DM2, increased body mass index (BMI) and increased waist-to-hip ratio (C/C) were the risk factors identified in all the participating countries, the last three being the most important, associated with an increased risk of presenting with DM2 (3.15, 2.76, and 3.63 times, respectively) ^18^. While these risk factors are universal, this study showed that amongst individuals with BMI less than 21, the prevalence of DM2 was 6 times higher in residents of low-income countries compared to those residing in high-income countries. Interestingly, this higher prevalence of DM2 at lower BMI levels in individuals from low-income countries was also observed in the rates of progression of prediabetes to DM2. Thus, while an annual progression of 11% was demonstrated in the Diabetes Prevention Program (DPP) in the US ^19^ and 6% in the Finnish Diabetes Prevention Study (DPS) ^20^, while the Indian Diabetes Prevention Program (IDPP) showed an annual progression of 18% ^21^. In Colombia ^22^ an annual progression of 9% was reported.

**Figure 1:**
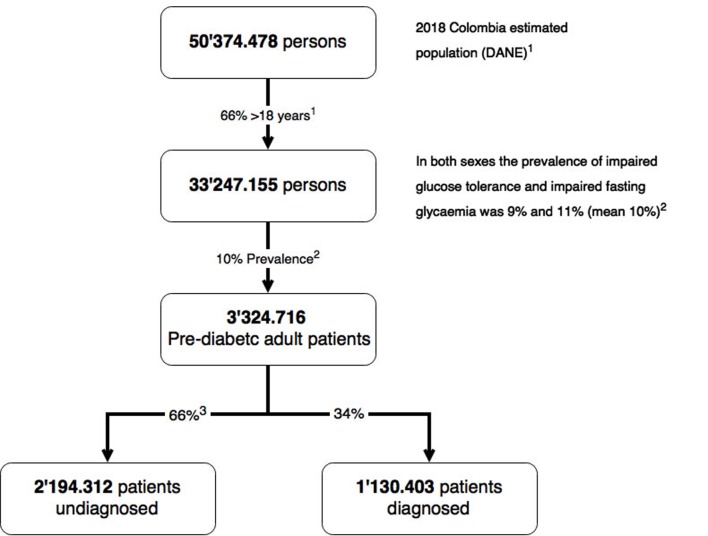
Estimated number of pre-diabetic subjects in Colombia 2018 ^8,15,21^

Based on the projected population of Colombia in 2018 ^23^, there will be 3.2 million adult pre-diabetic patients, of which 2.1 million will not be diagnosed or treated, that is, two out of every three Colombian prediabetics will not be protected from the risk of becoming diabetic or suffering cardio-vascular events ^11^ (Fig. 1). The importance of the identification of individuals with prediabetes lies in the possibility that their early management could arrest the growth in the incidence of DM2 that is currently occurring ^24^, especially in low- and middle-income countries ^4^.

Recent data from the IDF ^4^ show that the number of people with DM2 has risen from 135 million in 1995 to 415 million in 2015, with the prevalence rising more rapidly in low- to middle-income countries, and estimates suggest that by 2040 there will be 642 million diabetic patients, an increase of 64% in the diabetic population. In 2015, 5 million deaths were attributed to DM2, 43% of which occurred were patients under 50 years of age ^4^. In addition, DM2 is the major cause of blindness, renal failure, myocardial infarction, cerebrovascular accident and non-traumatic lower limb amputation, complications that are partly the result of the lack of an early diagnosis and a timely intervention to control the disease from the prediabetes state ^25^ ^,^ ^26^.

Prediabetes, besides being an important risk factor for the development of DM2, is also a risk factor for CVD. It has been shown that in the prediabetic state there is already vascular damage, the severity of which depends on the time of the onset of hyperglycaemia, since chronically elevated glucose causes pan-vascular damage: macro and micro-angiopathy, due to two mechanisms inherent to atherosclerotic disease: oxidation and vascular inflammation ^27^ ^-^ ^31^. In prediabetes these phenomena already coexist, and when DM2 is diagnosed years later, the vascular damage has already been magnified via the Metabolic Memory (MM) mechanism, in which oxidation of the cytochrome chain in the mitochondria transform its production of ATP in reactive oxygen species, which leads to apoptosis of endothelial cells and irreversible damage to the vascular wall ^28^ ^-^ ^31^. Hence, the importance of controlling hyperglycaemia from the onset of prediabetes is related to avoiding vascular damage ^32^ and its perpetuation through MM ^29^.

The time spent waiting for hyperglycaemia to reach the currently accepted cut-off levels for the diagnosis of DM2 and to intervene, may allow vascular damage to advance and become irreversible. This is demonstrated in the majority of clinical trials in patients with DM2 with more than four years of evolution for whom intensified therapies or new hypoglycaemic drugs, have had no effect in decreasing CV events ^33^ ^,^ ^34^ despite reaching the recommended levels of HbA1C, in contrast to the UKPDS intervention study in patients with a recent diagnosis of DM2 who showed a reduction in CV events ^35^ ^,^ ^36^.

From a physiological perspective, it is known that there are cells that do not have the capacity to regulate the transport of glucose in the presence of hyperglycaemia, hence DM2 complications occur in retina, mesangial cells and neurons, the three types of cells that do not adapt to exposure to this state ^31^. Damage to these cells begins when fasting blood glucose levels are still normal but there are already extensive post-prandial hyperglycaemic peaks ^37^, leading to the proposal that the crucial mechanism for vascular damage is insulin resistance, which is characterized by a close connection between hyperglycemia, increased dense and small LDL, vascular endothelial dysfunction, morphological alterations of the vascular wall and coagulation. all of which explains why the patient with chronic hyperglycaemia presents atherothrombotic outcomes more frequently through all these factors which contribute to the major vascular problem of the diabetic patient - Early Vascular Aging Syndrome (EVAS). This syndrome starts developing at the onset of insulin resistance, manifesting as arterial stiffness, increased pulse wave velocity, increased rebound wave with a consequent increased augmentation index, increase in central pressure and central pulse pressure, alterations which all appear long before arterial hypertension measured with traditional brachial blood pressure presents ^38^ ^,^ ^39^. Therefore, it is important to detect the onset of this process early to avoid brain-cardio-renal-angio-vascular complications. However, currently in our health system the detection and treatment of prediabetes is not a common approach. The Cardio-Metabolic and Diabetes-Hypertension pair are time bombs, and triggers for early CV outcomes, a situation aggravated by the mismanagement or non-comprehensive management of all risk factors, necessary approaches to preserve or restore adequate functionality of the vascular wall. In conclusion, the lack of diagnosis and treatment of prediabetes are important factors in prevention ^40^.

## Identification of subjects at risk of Prediabetes and DM2

Risk scales are useful questionnaire based tools, which allow a more cost-effective model for screening for various diseases. For the identification of subjects at risk of Prediabetes and DM2, the Finnish Diabetes Risk Score (FINDRIS) has proven to be simple, quick, economical, non-invasive and reliable and has been evaluated in several countries of differing income, which have shown different cut-off points associated with risk, as well as differences in sensitivity and specificity ^41^ ^,^ ^42^.

The FINDRIS which does not require laboratory tests, is a questionnaire of 8 easy to answer questions to determine the presence of risk factors for DM2 identified in several populations: age, BMI, physical activity, fruit and vegetable intake, medical treatment of hypertension, history of hyperglycaemia and family history of diabetes (Table 3). The answers generate a score for each of the risk factors, with the total sum of the points classifying an individual's risk of developing DM2 in the next 10 years as low, moderate, high and very high. For the Finnish population, the questionnaire cut-off points proposed were as follows ^41^:

• Risk score from 0 to 14 points indicates a low or moderate risk of diabetes (1-17% chance of DM2 in 10 years).
• Risk score of 15-20 points indicates a high risk of diabetes (33% chance of DM2 in 10 years).
• Risk score> 20 points indicates a very high risk of diabetes (50% chance of DM2 in 10 years).

**Table 3:** FINDRISC risk scale validated in Colombia. FINDRISC test for diabetes screening and other glucose regulation abnormalities 54

Name	Phone
1. Age. years	⎕Less than 45 years (0 p.)
	⎕ 45-54 years (2 p.)
	⎕ 55-64 years (3 p.)
	⎕ More than 64 years (4 p.)
2. Body Mass Index:	Weight. Kg
	Height. m
BMI	⎕ Less than 25 kg/m² (0 p.)
	⎕ Between 25-30 kg/m² (1 p.)
	⎕ More than 30 kg/m² (3 p.)
(It is calculated by dividing weight in kilos by height in square meters). E.g.: Weight 70 kg/Height 1.70 m^2^ = 70/2.89 = 24.2 kg/m^2)^	
3. Waist circumference. cm	Pass measuring tape in the middle of both side costal margin and iliac crest and completely horizontal (although it does not pass through the umbilicus). The person must breathe normally a couple of times, then the smallest measurement is taken (when the person exhales the air)
Men	⎕ Less than 94 cm (0 p.)
	⎕ Equal to or greater 94 cm (4 p.)
Women	⎕ Less than 90 cm (0 p.)
	⎕ Equal to or greater 90 cm (4 p.)
4. Are you physically active for at least 30 minutes daily, at work and/or in your free time?:	⎕ Yes (0 p.)
	⎕ No (2 p.)
5. How often do you eat vegetables or fruit?:	⎕ Every day (0 p.)
	⎕ Not every day (1 p.)
6. . Have you ever taken medications for high blood pressure on a regular basis?:	⎕ No (0 p.)
	⎕ Yes (2 p.)
7. Have you ever been found to have high blood glucose?	(e.g. in a health examination, during an illness, during pregnancy)
	⎕ No (0 p.)
	⎕ Yes (5 p.)
8. Have any members of your immediate family or other relatives been diagnosed with diabetes (type 1 or type 2)?	⎕ Yes: parents, siblings or children (5 p.)
	⎕ Yes: grandparents, uncle, aunt, ﬁrst cousin (3 p.)
	⎕ Other relatives or none (0 p.)
(Note: Also accounts a diabetes "by age" or "when was already old")	
Total risk score	
Add up the points for answered questions, to know your risk.	
Total score (maximum 26 p).	
Interpretation: If the test score is equal to or greater than 12, you have high probability of having diabetes or other abnormality of glucose regulation.		

Adapted from reference 54: Clinical practice guide for the diagnosis, treatment and follow-up of type 2 diabetes mellitus in the population over 18 years of age. General Social Security Health System - Colombia.

As the recommendation of those who developed this questionnaire ^41^ ^,^ ^42^ was to validate the instrument for use in each country, in Colombia ^43^ we conducted a population study to evaluate the FINDRIS questionnaire and establish the scores associated with increased risk of DM2 in our population. It was demonstrated that FINDRIS is a useful screening tool to identify subjects with unknown DM2 and to predict the incidence of DM2 among prediabetics and the cutoff point for predicting DM2 in prediabetics was 13 in men and 16 in women ^43^. Another group ^9^ implemented the ColDRIS study, also to develop a risk rating model for the Colombian population, and to assess the capacity of the ColDRIS to detect undiagnosed DM2. The ColDRIS questionnaire was adapted from the FINDRIS and validated in the Colombian population, with a change in the cut point only for abdominal circumference, identifying that a score equal or greater of 12 detected individuals at risk of DM2 with a sensitivity of 74% and a specificity of 60%. These values ​​suggest that the number of false negatives is low, and according to the literature evidence these patients will not present with complications in the subsequent three years, at which time re-screening is recommended. The shorter ColDRIS questionnaire consists of four variables: age, waist circumference, use of medications for blood pressure control and personal history of diabetes, and unlike FINDRIS does not consider diet, sedentary lifestyle, glucose history and BMI.

As the two studies conducted in Colombia show that the FINDRIS can be used as a simple, safe and no-cost test useful in identifying people at high risk of developing DM2, the Consensus group recommends that necessary efforts should be made to introduce the survey FINDRIS universally at the primary care level, allowing the level of risk of a patient to be defined before requesting costly laboratory tests. Amongst these tests, assessment of HbA1C as a diagnostic test for prediabetes or DM2 is controversial as evidence for its utility is inconsistent in terms of enhancing the sensitivity and / or specificity of the identification of individuals with prediabetes or DM2. It has also been reported that it is not a better diagnostic tool than fasting blood glucose or the OGTT ^44^ ^-^ ^50^. A recent analysis in Colombian adults, suggests that this test should be used only for the follow-up of patients in which the effect of the treatments implemented are evaluated, rather than for screening, in order to reduce costs to the health system ^44^.

**Figure 2:**
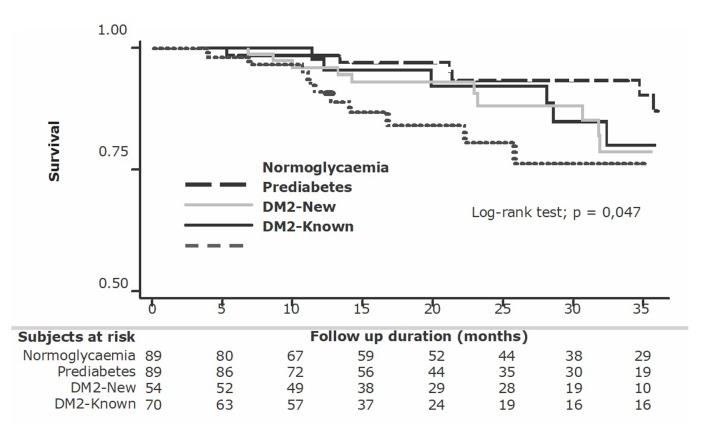
Colombian patients' survival after the ﬁrst myocardial infarction by blood glucose status evaluated at hospital discharge

For the Consensus the most appropriate diagnostic test for the diagnosis of prediabetes is the measurement of fasting plasma glucose and in case of doubtful results, confirmation with an OGTT, tests that must be requested if, as proposed in the Colombian Guide of Practice Clinic for the diagnosis, treatment and follow-up of DM2 in the population over 18 years of age, the FINDRIS score in adults is equal or greater than 12 (strong recommendation in favour, quality of the evidence: moderate). In Figure 2 an algorithm for the evaluation and management of the risk of glycaemic alterations is proposed.

## What do the Guidelines say about Prediabetes?

The Clinical Practice Guideline of Colombia for diagnosis, treatment and monitoring of type 2 diabetes mellitus in the population over 18 years / 2015 Guide No. GPC-2015-51, recognizes the terms increased risk of diabetes or prediabetes, recommending that people with a score equal to or greater than 12 on the Findrisk scale but do not meet diagnostic criteria for DM2, should establish the presence of increased risk categories of diabetes for inclusion in DM2 prevention programs ^51^. Characteristics of individuals with an increased risk of diabetes are altered IFG; between 100 and 125 mg/dL and/or glucose intolerance (IGT) with glycaemia 2 hours post 75 g oral glucose load between 140 and 199 mg/dL.

The Guidelines of the Latin American Association of Diabetes (ALAD) on the diagnosis, control and treatment of DM2 with Evidence-Based Medicine also recognizes the term prediabetes and explains the criteria for the diagnosis of DM2 and disorders of the regulation of glucose ^52^ ^,^ ^53^. This guide uses the values ​​previously stated for IFG and IGT, but also considers HbA1c values ​​between 5.7 and 6.4% as a diagnostic criterion for prediabetes, as does the new 2017 ADA guidelines ^1^.

The Consensus of the American Association of Endocrinology and the American College of Endocrinology published an algorithm to treat patients with Prediabetes, always beginning with lifestyle changes. The most important parameters to consider are the assessment of CV risk factors, measures to normalize weight and the treatment of hyperglycaemia to normalize IFG or ITG ^54^.

The Guidelines of the American Diabetes Association (ADA) 2017 established the criteria to evaluate the presence of DM2 and Prediabetes in asymptomatic adults and established the risk factors detailed in Table 3 ^1^.

The Guidelines of the European Society of Cardiology in collaboration with the European Association for the Study of Diabetes, published some recommendations around DM2, Prediabetes and Cardiovascular Diseases, establishing guidelines for the timely diagnosis of Diabetes or Prediabetes in patients with Cardiovascular Disease (CVD). They recommend screening for patients with CVD for DM2, based primarily on HbA1c and fasting blood glucose and performing an OGTT when HbA1c or fasting blood glucose levels are inconclusive ^55^.

In its 2006 report, the World Health Organization (WHO) recognizes intermediate hyperglycaemia and indicates that it can be diagnosed with an altered glucose tolerance test result (of between 140 and 200 mg/dL) and/ or an altered fasting glycemia (of between 110 and 125 mg/dL) ^3^.

## Management of prediabetes for the prevention of DM2 and cardiovascular events

**Figure 3:**
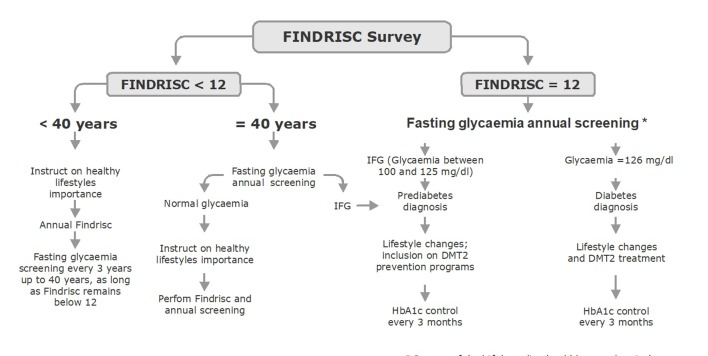
Risk and management assessment algorithm of Glycaemia disturbances

Several studies and meta-analyses have shown that the risk of CVD increases with blood glucose values ​​in the prediabetes range and that the increase in HbA1C in prediabetes values ​​is not only associated with greater progression to DM2, but also with an increased risk of CVD ^56^ ^-^ ^61^. A study in Colombia showed that hyperglycaemia was associated with a greater number of adverse outcomes in 439 individuals who survived a first acute myocardial infarction (AMI). Thus, 70% of the individuals who had an AMI had hyperglycaemia, but only the 20% were known diabetics with a long history of evolution; in 20% of the patients the diagnosis of DM2 was made when the infarct occurred and 30% of these patients were prediabetic ^62^. During a three-year follow-up period, the survival of patients with normal blood glucose was higher than that of patients with impaired glucose regardless of whether they were long-term or recently diagnosed diabetics or prediabetics. As shown in Figure 3, in the first months following MI, mortality in patients with DM2 and a longer duration of hyperglycaemia were those with the lowest survival rates, despite receiving the most intense pharmacological therapy. At the end of the three-year follow-up, the survival rates of the three groups with impaired glucose metabolism were similar and lower than that of the normoglycemic groups, demonstrating that hyperglycaemia is a factor that increases the risk of mortality, independently of whether their glucose levels defined them as prediabetic or diabetic. However, while patients with DM2 received hypoglycaemic treatment according to the guidelines, prediabetics, according to these same guidelines, did not receive metformin, which is the first-line pharmacological treatment for the management of hyperglycaemia, so we do not have data in these Colombian patients to determine whether management with metformin in the prediabetics could have increased their survival rate. However, UKPDS data in patients with recent-onset DM2 who received metformin had fewer CV events ^35^ ^,^ ^36^, which suggests that our prediabetic patients may also have benefited from metformin treatment not only via a decrease the progression from prediabetes to DM2, but also by a reduction in CV events. These data have served to question the utility of the blood glucose cut-off points currently used in low- and middle-income countries for the diagnosis of DM2, which were based on a Finnish study that evaluated the association of glycemia with retinopathy, not CV outcomes ^63^. A research group in London ^64^ ^,^ ^65^ elegantly demonstrated the "point of no return" during the course of peripheral neuropathy, cardiomyopathy, and diabetic nephropathy, when chronic hyperglycaemia causes functional alterations and structural alterations. In an experimental model of diabetes caused by the administration of streptozotocin, a substance that destroys pancreatic beta cells in rats, they observed that if the control of hyperglycaemia with insulin begins immediately and up to four weeks after induced diabetes, structural lesions in the nitrergic nerves are prevented. However, if treatment begins after 8 weeks of hyperglycaemia, the administration of insulin normalizes the glycaemia, but does not reverse the structural changes that manifest in the micro and macro complications vascular diseases of diabetes. Therefore, as proposed for several years ^66^, in order to achieve positive results with primary cardiovascular prevention in patients with hyperglycaemia, it is necessary to begin to implement control once prediabetes is detected and the associated risk factors, such as overweight and obesity , hypertension and dislipidemia are present, via both changes in lifestyle habits, as well as pharmaceutical therapy with drugs such as metformin, statins and inhibitors of the renin-angiotensin-aldosterone system (RAAS).

Recently, the HOPE three studies showed that the administration of these last two drugs reduced the relative risk of CVD in intermediate-risk patients by 40% ^67^ ^-^ ^69^. Most clinical studies show that when the patient is already a long-term diabetic and the damage caused by hyperglycaemia in the vascular system and other tissues is present, there is a failure to prevent outcomes due to macrovascular complications either with intensified treatment or by the use of new hypoglycaemic agents ^33^ ^,^ ^34^. At which time, as demonstrated by the STENO study, the most important objective is the rigorous control of blood pressure and lipids, because of late attempts to strictly control glycemia ^66^. The critical factor in the prevention of CVD associated with hyperglycaemia is not the magnitude of HbA1c is lowering, but rather the time at which the control of hyperglycaemia begins, a concept that is also related to the pathophysiology of vascular complications of diabetes. Evidence demonstrates the important role played by advanced glycation end products (AGEs-) in the development of these complications ^70^ and the longer the period of hyperglycaemia, the greater the formation of these products and the greater degree of irreversible glycosylation of structural proteins of the cell membranes they produce. If Metformin is used in time, it may reduce the structural and functional changes in various tissues and systems mediated by AGEs. In a canine model of diabetes, it was shown that four months of treatment with metformin significantly reduced both myocardial stiffness and glycosylated collagen content, showing that metformin can control the deleterious changes associated with protein glycation *in vivo* ^70^.

Considering this background, the Consensus suggests that the laboratories which commercialize metformin carry out the necessary steps to obtain the approval by the National Institute for Surveillance of Drugs and Foods (INVIMA), the regulatory agency for medicines in Colombia, for the prescription of metformin as adjuvant treatment to the therapeutic changes in lifestyle habits for the management of prediabetes, as has already been done in several developed countries such as the United Kingdom and in some Latin American countries such as Peru and Mexico. Following approval, the prescription of metformin for the management of prediabetes should be disseminated through continuing medical education programs.

We emphasize, that exercise, healthy diet and weight loss are important measures for the management of prediabetes. However, also having a pharmacological tool that helps reduce blood glucose and both progression to DM2, and macro and microvascular complications, but who's use in Colombia has been limited by physician's lack of knowledge of the importance of diagnosis and management of prediabetes as well as by it not having regulatory body approval as a treatment adjuvant for prediabetes, is a measure that should be implemented according to the recommendations of several guides reviewed here.

How the progression from prediabetes to DM2 can be prevented is an important topic both for academic organizations globally and for the entities in charge of public health in our country ^71^. Several studies on lifestyle intervention and / or metformin have shown a sustained reduction in the rate of progression of prediabetes to DM2, thus the Diabetes Prevention Study (DPS), Finnish work ^20^ performed in a population at high risk for diabetes; with glucose intolerance, a direct family history of DM2, overweight (BMI ≥ 25 kg / m^2)^ and aged between 40 and 65 years, included 523 people who were randomized to receive intervention with lifestyle changes (diet and exercise) designed to achieve with a loss weight of 5% or more, or a control group. The cumulative incidence of diabetes after 4 years was 11% in the intervention group and 23% in the control group and the risk of diabetes was reduced by 58% (*p* <0.001) in the intervention group during the study. The reduction in the incidence of diabetes was directly associated with changes in lifestyle. The study concluded that DM2 can be prevented with changes in lifestyle in high-risk subjects. The Diabetes Prevention Program (DPP) study conducted in the USA ^19^ was a randomized, multicentre clinical trial in patients over 25 years old with a BMI greater than 24, impaired fasting blood glucose and glucose intolerance, followed up for an average of 2.8 years. 3,234 patients (68% women) were randomized to receive one of three interventions: an intensive lifestyle modification program (n = 1,078), standard lifestyle recommendations plus metformin 850mg twice daily (n = 1,073) or standard lifestyle recommendations plus placebo (n = 1,082). Progression to DM2 was 4.8 case / 100 person-years in the intensive lifestyle changes intervention group, 7.8 in the metformin group and 11.0 in the placebo group. The intensive lifestyle intervention and metformin groups had 58% and 31% lower incidence respectively, when compared to the placebo group. The study demonstrated that an intensive lifestyle modification program, as well as treatment with metformin are effective in preventing or delaying the incidence of DM2 in a high-risk population. The improvements in the lifestyle intervention + metformin groups were more robust in people older than 60 years and in those with a lower body mass index, while within the metformin only group results were better in younger people and in those with a a higher body mass index (≥ 35). This cohort were followed up at 10 years to evaluate the incidence of DM2 and the evolution of weight loss ^72^, of 2,766 of the patients included in the original study, 910 of the intensive lifestyle changes group, 924 of the metformin group and 932 of the placebo group were included. At the 10 years of follow-up, the incidence of DM2 was 34% lower in the intensive lifestyle changes group and by 18% in the metformin group compared with the control group. The specific analysis of the follow-up phase after the end of the original study showed a progression from prediabetes to DM2 of 5.9% person-years in the intensive lifestyle changes group, 4.9% person-years in the metformin group and 5.6% person-years for the placebo group. Therefore, metformin decreased the progression to new cases of DM2 by 13% relative to the placebo group, while in the intensive lifestyle changes group, progression to DM2 was 5% higher than in the control group. The best results obtained with metformin in the long-term follow-up in relation to the group of intensive changes in lifestyle are explained by the gradual and gradual weight gain that was observed in this group, confirming the practical difficulty of maintaining the Weight loss and control of hyperglycaemia with changes in long-term lifestyle.

This follow-up study continued, to evaluate not only the progression to DM2 but also the long-term effects of the lifestyle changes intervention and metformin on the development of microvascular complications after 15 years of follow-up, a phase known as the DPP Outcomes Study (DPPOS) ^73^. After 15 years of intervention, the average annual incidence of diabetes was 7.0%, 5.7%, and 5.2% in the placebo, metformin, and lifestyle intervention groups, respectively. The incidence of diabetes was 27% lower in the lifestyle intervention group and 18% lower in the metformin group compared to the placebo group. The cumulative incidence of diabetes at 15 years was 55% in the lifestyle change group, 56% in the metformin group, and 62% in the control group. At the end of the study, the prevalence of microvascular complications was higher in the placebo group, but there were no significant differences between the 2 treatment groups. In addition to the lower rate of progression to DM2 in the DPPOS cohort, other benefits of the lifestyle intervention and metformin were reported, such as the reduction of risk factors for CVD, reduction in the prevalence of lower tract symptoms urinary associated with obesity and diabetes, and improvements in the quality of life.

In cost-effectiveness ratio analysis, 10 years after the implementation of lifestyle changes or metformin for the prevention of diabetes using an intention-to-treat analysis of the DPP / DPPOS studies ^74^, it was concluded that lifestyle changes were cost-effective, as was metformin compared to the control group and that there was a good cost-effectiveness ratio of investment in lifestyle change interventions and metformin to prevent the progression of prediabetes to DM2 in high-risk adults. Thus, it was estimated that the direct cumulative medical costs per capita, without a discount of interventions as implemented in the DPP, were greater for the lifestyle (4,572 US dollars) than for metformin (2,281 US dollars) or for the control group ($ 769 US dollars). When a discount was considered for the lifestyle changes group, based on the fact that during the 3-year period of the DPP the patients received training in sessions of 10 participants, the per capita costs were 2,995 US dollars, which shows that at least in the USA, to reduce the progression of prediabetes to DM2, it is more expensive to induce changes in lifestyle than to administer metformin.

The DPP gave rise to a similar protocol implemented in India known as the Indian Diabetes Prevention Program (IDPP) ^21^ that included 402 prediabetics, a study that confirmed that lifestyle changes and metformin prevent DM2 to a similar degree, but without synergistic effects when implemented together. However, it should be noted that the IDPP patients were on average 5 years younger, had a 10 cm lower abdominal circumference and a BMI 8 points lower than the Americans in the DPP, and also that the average fasting and post-load glycemia were slightly lower (Table 3). The comparison of these two studies ^75^ clearly demonstrates the greater sensitivity of populations of low- and middle-income countries to develop DM2 at lower levels of visceral adiposity and BMI, as recently confirmed by the PURE study ^18^, and also the better response to a metformin intervention; thus the rate of progression from prediabetes to DM2 was 18.3% per year in Asian-Indians, while in North Americans it was 11.0% and in Finnish DPS it was only 6.0%. In addition, metformin at a dose of 500 mg / day was more effective in reducing the progression of prediabetes to DM2 in Asian-Indians, with an annual reduction of 14.5%, while in Americans in whom 1,700 mg / day was used, the annual reduction was only 7.2%, which means that in the population of a low-income country like India, the number needed to be treated to prevent a case of DM2 is 6.9, while in the US it rises to 13.9.

**Table 4:** Characteristics comparison of subjects included in three DM2 prevention studies

	DPP			IDPP			DPS	
Characteristics	Placebo (n: 1,082)	Metformina (n: 1,073)	Lifestyle change (n: 1,079)	Control (n: 136)	Metformina (n:133)	Lifestyle change (n: 133)	Control (n: 257)	Lifestyle change (n: 265)
Gender								
Man	335	363	345	104	104	107	81	91
Woman	747	710	734	32	29	26	176	174
Age (years)	50.3 ± 10.4	50.9 ± 10.3	50.6 ± 11.3	45.2 ± 5.7	45.9 ± 5.9	46.1 ± 5.7	55.0 ± 7.0	55±7
BMI (Kg/m^2)^	34.2 ± 6.7	33.9 ± 6.6	33.9 ± 6.8	26.3 ± 3.7	25.6 ± 3.7	25.7 ± 3.3	31.0 ± 4.5	31.3±46
Abdominal girth (cm)	105.2 ±14.3	104.9 ± 14.4	105.1 ± 14.8	90.8 ± 7.5	89.7 ± 9.5	89.0 ± 7.9	100.5 ± 10.9	102.0 ± 11.0
Serum glucose (mg/dL)								
Fasting	106.1 ± 8.4	106.5 ± 8.5	106.3 ± 8.1	99.1 ± 14.4	97.3 ± 14.4	97.3 ± 12.6	110.0 ± 13.0	109.0 ± 14.0
Glucose tolerance oral test	164.5 ± 17.1	165.1 ± 17.2	164.4 ± 16.8	155.0 ± 12.6	153.2 ± 12.6	153.2 ± 12.6	159.0 ± 26.0	159.0 ± 27.0

Mean values +/- Standard Deviation

BMI: Body Mass Index

Adapted from reference 75

A recent and extensive review ^76^ on the use of metformin in Prediabetes and in Diabetes Prevention, concluded that there is sufficient evidence to support the therapeutic use of metformin for the prevention of diabetes and mentions that interventions in the lifestyle are difficult for patients to maintain; the weight lost tends to be gained again and at a greater rate over time, while metformin is effective and safe for delaying or preventing the onset of DM2. In addition, decades of clinical use of metformin in patients with DM2 have shown that metformin is generally well tolerated and safe.

## Particularities related to the increase in Prediabetes and DM2 in Colombia

The analysis of the DPS, DPP and IDPP studies ^75^ plus the epidemiological and clinical observations made in our environment of the rapid growth of visceral adiposity and DM2 ^77^ ^,^ ^78^ led us to propose that the epigenetic mechanism of fetal programming is an influence in the low social strata, where pregnant mothers with inadequate protein intake have higher rates of offspring with intrauterine growth retardation and low birth weight who will in the future be exposed to increasing levels of ultra-processed carbohydrates in the diet. In adulthood, these low birth weight offspring have greater sensitivity to develop insulin resistance, low-grade inflammation and DM2 at lower levels of visceral adiposity, linked to the lower amount of muscle mass and strength consequent to the poor nutritional intake of the mother during pregnancy ^79^ ^,^ ^80^. In support of this proposal, we have noted a higher concentration of C-reactive protein (CRP), a marker of low-grade inflammation, in children in Bucaramanga, Colombia than that observed in American and European children of the same age and the same BMI. ^81^. We have also observed in Colombian children with low birth weight for gestational age evaluated at 10 years old, that those in the highest quartile of BMI were those with the highest CRP concentrations, had higher HOMA index of insulin resistance and a larger amount of fat mass but lower handgrip strength, and a higher metabolic risk index ^82^. Low handgrip strength is a known risk factor for cardiovascular and all-cause mortality in adults in the PURE study ^83^, and in patients with Prediabetes and DM2 of the ORIGIN study ^84^. There is evidence of clear differences in handgrip strength between populations, whereby low and middle income countries, have lower strength than people in high income countries ^85^. Indeed, the important role of muscle mass and strength in the regulation of systemic inflammation and insulin sensitivity has recently been increasingly highlighted ^86^, given the cross-talk between muscle and fat mass in the maintenance of an adequate metabolic balance. Alterations in this balance begin during intrauterine life, as proposed by Baker a number of years ago in highlighting the importance of fetal programming and its dependence on maternal nutrition ^87^. In practical terms, these results demonstrate the need for the prediabetic patient to prevent progression to DM2 to undertake a physical activity program that includes strength, as well as cardio-respiratory exercise ^88^, particularly in low-income country populations. The environmental and socio-economic factors in these populations predispose them to a greater sensitivity to develop diseases associated with insulin resistance such as DM2, CVD and non-alcoholic fatty liver ^89^ ^,^ ^90^ through adaptive epigenetic mechanisms related to the synthesis of substances involved in the regulation of food intake, insulin sensitivity and systemic inflammation ^91^ ^-^ ^95^.

## Conclusions and recommendations

1. The prevalence of Prediabetes in Colombia is high, and despite being recognized and categorized in the main national and international medical guides, and being included in the International Classification of Diseases (ICD-10) in Colombia, knowledge and awareness of health professionals and in the community is limited. It is therefore recommended that the Faculties of Health Sciences and Scientific Associations related to continuing medical education implement actions aimed at improving the knowledge of the health professionals and the community in general in relation to identifying, diagnosing and treating patients with prediabetes. The group of experts also recommends that the educational programs when implemented should emphasize the need for a global risk approach, including the importance of awareness and evaluation of other risk factors such as family history of DM2, overweight and obesity, dislipidemia and hypertension. The Ministry of Health must make disseminate the code for prediabetes and disclose it so that the disease can appear as a specific diagnosis in the patient's medical records.

2. The two studies carried out in Colombia demonstrate the usefulness of the FINDRIS survey in identifying subjects at risk of prediabetes and DM2; and it is therefore recommended that the FINDRIS should be applied as a matter of government policy to all the healthcare promotion and healthcare institutions in the country. All adult patients should complete the form before a consultation, with any non-medical member of the health team.

The application of FINDRIS will avoid unnecessary laboratory tests and thus generate a significant saving in health spending, so it is essential that a greater dissemination of this tool is implemented as a requirement, carried out at the Primary Care level.

To determine whether to use the complete FINDRIS or the shortened version in Colombia, it is necessary to define the objectives of the evaluation: in terms of broad public health programs, using the short form may help to save time, however, from the clinical point of view, using the shortened questionnaire reduces the potential for making comparisons with other evaluations made using the original FINDRIS. The Consensus therefore recommends using either of the two versions to assess the risk of the patients and insists that the application of FINDRISC must be an institutional policy that should be implemented even when the patient arrives at the consultation with the result of fasting blood glucose.

3. There is a large body of evidence showing that Prediabetes progresses towards DM2 at an annual rate of at least 10% and that independent of progression to DM2, prediabetes is a risk factor for cardiovascular outcomes. On this basis, the Committee recommends that once detected and diagnosed, immediate management should be initiated through lifestyle changes, and assessment repeated at 3 and 6 months. If the patient does not achieve a weight loss of at least 5% and HbA1C values ​​are not normalized, pharmacological management should begin; starting with a dose of 500 mg / day of metformin, increasing to 1,500 - 1,700 mg/day, according to tolerance.

## References

[1] American Diabetes Association (2016). Standars of medical care in diabetes. Diabetes Care.

[2] López-Jaramillo P, Nieto-Martínez RE, Aure-Fariñez G, Mendivil CO, Lahsen RA, Silva-Filho RL (2017). Identification and management of prediabetes results of the Latin America strategic prediabetes meeting. Rev Panam Salud Publica.

[3] WHO (2006). Definition and diagnosis of diabetes mellitus and intermediate hyperglycemia.

[4] International Diabetes Federation (2015). Diabetes Atlas, 7th edition.

[5] Instituto Colombiano de Bienestar Familiar (2010). Encuesta Nacional de la Situación Nutricional en Colombia (ENSIN)..

[6] Clarke WL, Cox D, Gender-Frederick LA, Cater W, Pohl SL (1987). Evaluating clinical accuracy of systems for self-monitoring of blood glucose. Diabetes Care.

[7] Instituto Colombiano de Bienestar Familiar (2005). Encuesta Nacional de la Situación Nutricional en Colombia (ENSIN).

[8] Camacho PA, Gomez-Arbelaez D, Molina DI, Sanchez G, Arcos E, Narvaez C (2016). Social disparities explain differences in hypertension prevalence, detection and control in Colombia. J Hypertens.

[9] Barengo NC, Tamayo DC, Tono T, Tuomilehto J (2017). A Colombian diabetes risk score for detecting undiagnosed diabetes and impaired glucose regulation. Prim Care Diabetes.

[10] Lopez-Jaramillo P, Sanchez G, Perez M, Calderon JE, Sotomayor A, Suarez M (2011). Colombo-Ecuadorian study to determinate the prevalence of pre-diabetes in patients with a first acute myocardial infarction. J Diabetes.

[11] Rafael G, Gonzalez-Villalpando C, Lopez-Jaramillo P, Acosta T, López RR, R Nieto-Martínez, Bergman M (2014). Prediabetes and Diabetes Prevention Initiatives in Latin America (LA). Global health perspectives in prediabetes and diabetes prevention.

[12] Lopez-Jaramillo P, Sanchez R, Diaz M, Cobos L, Bryce A, Parra-Carrillo JZ (2013). Latin America consensus on hypertension in patients with diabetes type 2 and metabolic syndrome. J Hypertens.

[13] Tseng E, Greer RC, O&apos;Rourke P, Yeh HC, McGuire MM, Clark JM (2017). Survey of primary care providers&apos; knowledge of screening for, diagnosing and managing prediabetes. J Gen Intern Med.

[14] Garay J, Camacho P, Cohen D, Calderon C, Parra-Zuluaga G, Lopez-Jaramillo P (2017). Survey of knowledge for diagnosing and managing prediabetes in Latin-America. Diabetol Metab Syndr.

[15] Ministerio de Salud y Protección Social Codificación CIE10 para prediabetes.

[16] Figueroa FN, Morales J, Melgarejo A, Forero J, Motoa G, León JA (2011). Characterization of patients with pre-diabetes in first-level health care service institutions Cali, Colombia. Colomb Med (Cali).

[17] Kabadi UM (2017). Major pathophysiology in prediabetes and type 2 diabetes decreased insulin in lean and insulin resistance in obese. J Endocr Soc.

[18] Dagenais GR, Gerstein HC, Zhang X, McQueen M, Lear S, Lopez-Jaramillo P (2016). Variations in diabetes prevalence in low-, middle-,and high-income countries Results from the prospective urban and rural epidemiology study. Diabetes Care.

[19] Diabetes Prevention Program Research Group (2002). Reduction in the Incidence of Type 2 Diabetes with Lifestyle Intervention or Metformin. N Engl J Med.

[20] Tuomilehto J, Lindström J, Eriksson JG, Valle TT, Hämäläinen H, Ilanne-Parikka P (2001). Prevention of type 2 diabetes mellitus by changes in lifestyle among subjects with impaired glucose tolerance. N Engl J Med.

[21] Ramachandran A, Snehalatha C, Mary S, Mukesh B, Bhaskar AD, Vijay V, Indian Diabetes Prevention Programme (2006). The Indian Diabetes Prevention Programme shows that lifestyle modification and metformin prevent type 2 diabetes in Asian Indian subjects with impaired glucose tolerance (IDPP-1). Diabetologia.

[22] Gomez-Arbelaez D, Alvarado-Jurado L, Ayala-Castillo M, Forero-Naranjo L, Camacho PA, Lopez-Jaramillo P (2015). Evaluation of the Finnish Diabetes Risk Score to predict type 2 diabetes mellitus in a Colombian population A longitudinal observational study. World J Diabetes.

[23] Departamento Administrativo Nacional de Estadísticas (2005). Proyecciones de población.

[24] CDC (2017). National diabetes statistics report, 2017: estimates of diabetes and its burden in the United States.

[25] Shen J, Kondal D, Rubinstein A, Irazola V, Gutierrez L, Miranda JJ (2016). A Multiethnic Study of Pre-Diabetes and Diabetes in LMIC. Glob Heart.

[26] Bamberg F, Hetterich H, Rospleszcz S, Lorbeer R, Auweter SD, Schlett CL (2017). A Subclinical disease burden as assessed by whole-body MRI in subjects with prediabetes, subjects with diabetes, and normal control subjects from the general population The KORA-MRI Study. Diabetes.

[27] Goldberg RB (2009). Cytokine and Cytokine-like inflammation markers, endothelial dysfunction, and imbalanced coagulation in development of diabetes and its complications. J Clin Endocrinol Metab.

[28] Davis TM, Chubb SA, Bruce DG, Davis WA (2016). Metabolic memory and all-cause death in community-based patients with type 2 diabetes the Fremantle Diabetes Study. Diabetes Obes Metab.

[29] Ranjit Unnikrishnan I, Anjana RM, Mohan V (2011). Importance of controlling diabetes early--the concept of metabolic memory, legacy effect and the case for early insulinisation. J Assoc Physicians India.

[30] Misra A, Bloomgarden Z (2017). Metabolic memory evolving concepts. J Diabetes.

[31] Semenkovich CF (2017). We know more than we can tell about diabetes and vascular disease The 2016 Edwin Bierman Award Lecture. Diabetes.

[32] Garcia RG, Lopez-Jaramillo P (2008). Cardiovascular prevention in high-risk patients with type 2 diabetes mellitus when to start it?. Eur Heart J.

[33] Dluhy RG, McMahon GT (2008). Intensive glycemic control in the ACCORD and ADVANCE trials. N Engl J Med.

[34] Mannucci E, Monami M (2017). Cardiovascular safety of incretin-based therapies in type 2 diabetes Systematic review of integrated analyses and randomized controlled trials. Adv Ther.

[35] UK Prospective Diabetes Study Group (1998). Effect of intensive blood-glucose control with metformin on complications in overweight patients with type 2 diabetes (UKPDS 34). Lancet.

[36] Holman RR, Paul SK, Bethel MA, Matthews DR, Neil HA (2008). 10-year follow-up of intensive glucose control in type 2 diabetes. N Engl J Med.

[37] Mah E, Bruno RS (2012). Postprandial hyperglycemia on vascular endothelial function mechanisms and consequences. Nutr Res.

[38] Fortier C, Sidibé A, Desjardins MP, Marquis K, De Serres SA, Mac-Way F (2017). Aortic-brachial pulse wave velocity ratio A blood pressure-independent index of vascular aging. Hypertension.

[39] Barton M, Husmann M, Meyer MR (2016). Accelerated vascular aging as a paradigm for hypertensive vascular disease Prevention and therapy. Can J Cardiol.

[40] Qiu M, Shen W, Song X, Ju L, Tong W, Wang H (2015). Effects of prediabetes mellitus alone or plus hypertension on subsequent occurrence of cardiovascular disease and diabetes mellitus longitudinal study. Hypertension.

[41] Bergmann A, Li J, Wang L, Schulze J, Bornstein SR, Schwarz PE (2007). A simplified finnish diabetes risk score to predict type 2 diabetes risk and disease evolution in a German population. Horm Metab Res.

[42] Schwarz PE, Li J, Reimann M, Schutte AE, Bergmann A, Hanefeld M (2009). The Finnish Diabetes Risk Score is associated with insulin resistance and progression towards type 2 diabetes. J Clin Endocrinol Metab.

[43] Gomez-Arbelaez D, Alvarado-Jurado L, Ayala-Castillo M, Forero-Naranjo L, Camacho PA, Lopez-Jaramillo P (2015). Evaluation of the Finnish Diabetes Risk Score to predict type 2 diabetes mellitus in a Colombian population A longitudinal observational study. World J Diabetes.

[44] Lopez-Lopez J, Garay J, Wandurraga E, Camacho P, Higuera-Escalante F, Cohen D (2017). Glycosylated hemoglobin does not improve the detection of prediabetes and type 2 diabetes mellitus in colombian adults: a cross-sectional study. Plos One.

[45] Choi SH, Kim TH, Lim S, Park KS, Jang HC, Cho NH (2011). Hemoglobin A1c as a diagnostic tool for diabetes screening and new-onset diabetes prediction a 6-year community-based prospective study. Diabetes Care.

[46] Shibata K, Suzuki S, Sato J, Ohsawa I, Goto S, Iritani I (2005). Diagnostic accuracy of glycohemoglobin A1c (HbA1c) for postprandial hyperglycemia was equivalent to that of fasting blood glucose. J Clin Epidemiol.

[47] Bennett CM, Guo M, Dharmage SC (2007). HbA(1c) as a screening tool for detection of Type 2 diabetes a systematic review. Diabet Med.

[48] Cavagnolli G, Comerlato J, Comerlato C, Renz PB, Gross JL, Camargo JL (2011). HbA(1c) measurement for the diagnosis of diabetes is it enough?. Diabet Med.

[49] Kumaravel B, Bachmann MO, Murray N, Dhatariya K, Fenech M, John WG (2012). Use of haemoglobin A1c to detect impaired fasting glucose or Type 2 diabetes in a United Kingdom community based population. Diabetes Res Clin Pract.

[50] Lee H, Oh JY, Sung YA, Kim DJ, Kim SH, Kim SG (2013). Optimal hemoglobin A1C cutoff value for diagnosing type 2 diabetes mellitus in Korean adults. Diabetes Res Clin Pract.

[51] Aschner P, Muñoz O, Giron D, Garcia O, Fernandez-Avila D, L Casas (2016). Clinical practice guideline for the prevention, early detection, diagnosis, management and follow up of type 2 diabetes mellitus in adults. Colomb Med (Cali).

[52] ALAD (2013). Guias Latinoamericanas de Diabetes.

[53] Rosas-Saucedo J, Enrique CA, Brito-Córdova G, García-Bruce H, Costa-Gil J, Lyra R, Rosas-Guzmán J (2017). Consenso de Prediabetes Documento de Posición de la Asociación Latinoamericana de Diabetes (ALAD). Rev ALAD.

[54] Garber AJ, Abrahamson MJ, Barzilay JI, Blonde L, Bloomgarden ZT, Bush MA (2017). Consensus statement by the American Association of Clinical Endocrinologists and American College of Endocrinology on the comprehensivew type 2 diabetes management algorithm - 2017 executive summary. Endocr Pract.

[55] Rydén L, Grant PJ, Anker SD, Berne C, Cosentino F, Danchin N (2013). ESC Guidelines on diabetes, pre-diabetes, and cardiovascular diseases developed in collaboration with the EASD the Task Force on diabetes, pre-diabetes, and cardiovascular diseases of the European Society of Cardiology (ESC) and developed in collaboration with the European Association for the Study of Diabetes (EASD). Eur Heart J.

[56] Shaye K, Amir T, Shlomo S, Yechezkel S (2012). Fasting glucose levels within the high normal range predict cardiovascular outcome. Am Heart J.

[57] Eastwood SV, Tillin T, Sattar N, Forouhi NG, Hughes AD, Chaturvedi N (2015). Associations Between Prediabetes, by Three Different Diagnostic Criteria, and Incident CVD Differ in South Asians and Europeans. Diabetes Care.

[58] Huang Y, Cai X, Mai W, Li M, Hu Y (2016). Association between prediabetes and risk of cardiovascular disease and all cause mortality systematic review and meta-analysis. BMJ.

[59] Warren B, Pankow JS, Matsushita K, Punjabi NM, Daya NR, Grams M (2017). Comparative prognostic performance of definitions of prediabetes a prospective cohort analysis of the Atherosclerosis Risk in Communities (ARIC) study. Lancet Diabetes Endocrinol.

[60] Cefalu WT (2016). "Prediabetes": Are There Problems With This Label? No, We Need Heightened Awareness of This Condition!. Diabetes Care.

[61] Selvin E, Steffes MW, Zhu H, Matsushita K, Wagenknecht L, Pankow J (2010). Glycated hemoglobin, diabetes, and cardiovascular risk in nondiabetic adults. N Engl J Med.

[62] Gomez-Arbelaez D, Sánchez-Vallejo G, Perez M, Garcia RG, Arguello JF, Peñaherrera E (2016). Hiperglucemia se asocia a mayor número de desenlaces adversos en individuos latinoamericanos con infarto agudo de miocardio. Clin Investig Arterioscler.

[63] Lopez-Jaramillo P, Velandia- Carrillo C, Gomez-Arbelaez D, Aldana-Campos M (2014). Is the present cut-point to define type 2 diabetes appropiate in Latin-Americans. World J Diabetes.

[64] Cellek S, Foxwell NA, Moncada S (2003). Two phases of nitrergic neuropathy in streptozotocin-induced diabetic rats. Diabetes.

[65] Cellek S, Qu W, Schmidt AM, Moncada S (2004). Synergistic action of advanced glycation end products and endogenous nitric oxide leads to neuronal apoptosis in vitro a new insight into selective nitrergic neuropathy in diabetes. Diabetologia.

[66] Gaede P, Vedel P, Larsen N, Jensen GV, Parving HH, Pedersen O (2003). Multifactorial intervention and cardiovascular disease in patients with type 2 diabetes. N Engl J Med.

[67] Lonn EM, Bosch J, López-Jaramillo P, Zhu J, Liu L, Pais P (2016). Blood-Pressure Lowering in intermediate-Risk Persons without Cardiovascular Disease. N Engl J Med.

[68] Yusuf S, Bosch J, Dagenais G, Zhu J, Xavier D, Liu L (2016). Cholesterol Lowering in Intermediate-Risk Persons without Cardiovascular Disease. N Engl J Med.

[69] Yusuf S, Lonn E, Pais P, López-Jaramillo P, Zhu J, Xavier D (2016). Blood-pressure and cholesterol lowering in persons without cardiovascular disease. N Engl J Med.

[70] Jyothirmayi GN, Soni BJ, Masurekar M, Lyons M, Regan TJ (1998). Effects of Metformin on Collagen Glycation and Diastolic Dysfunction in Diabetic Myocardium. J Cardiovasc Pharmacol Ther.

[71] Barry E, Roberts S, Oke J, Vijayaraghavan S, Normasenll R, Greenhalgh T (2017). Efficacy and effectiveness of screen and treat policies in prevention of type 2 diabetes systematic review and meta-analysis of screening tests and interventions. BMJ.

[72] Knowler WC, Fowler SE, Hamman RF et al (2009). 10-year follow-up of diabetes incidence and weight loss in the Diabetes Prevention Program Outcomes Study. Lancet.

[73] Diabetes Prevention Program Research Group (2015). Long-term Effects of Lifestyle Intervention or Metformin on Diabetes Development and Microvascular Complications over 15-year follow-up the DPP Outcomes Study. Lancet Diabetes Endocrinol.

[74] Diabetes Prevention Program Research Group (2012). The 10-year cost-effectiveness of lifestyle intervention or metformin for diabetes prevention an intent-to-treat analysis of the DPP/DPPOS. Diabetes Care.

[75] Lopez-Jaramillo P (2008). Defining the research priorities to fight the burden of cardiovascular diseases in Latin America. J Hypertens.

[76] Hostalek U, Gwilt M, Hildemann S (2015). Therapeutic Use of Metformin in Prediabetes and Diabetes Prevention. Drugs.

[77] Lopez-Jaramillo P, Lahera V, Lopez-Lopez J (2011). Epidemic of cardiometabolic diseases A Latin American point of view. Ther Adv Cardiovasc Dis.

[78] Lopez-Jaramillo P, Rey JJ, Gomez-Arbelaez D, Rodriguez YA, Lopez-Lopez J (2011). Combatir la epidemia de diabetes mellitus tipo 2 en Latinoamerica caracteristicas especiales que demandan acciones innovadoras. Clin Invest Arterioscl.

[79] Lopez-Jaramillo P (2009). Cardiometabolic diseases in Latin America the role of fetal programming in response to maternal malnutrition. Rev Esp Cardiol.

[80] Lopez-Jaramillo P, Lopez-Lopez J (2010). Fetal programming and cardiometabolic diseases the role of angiotensin II and inflammation. Clin Invest Arterioscl.

[81] Lopez-Jaramillo P, Garcia G, Camacho P (2008). A, Herrera E, Castillo V Interrelationship between body mass index, C-reactive protein and blood pressure in a Hispanic pediatric population. Am J Hypertens.

[82] Cohen DD, Gomez-Arbelaez D, Camacho PA, Pinzon S, Hormiga C, Trejos-Suarez J, Duperly J, Lopez-Jaramillo P (2014). Low muscle strength is associated with metabolic risk factors in Colombian children The ACFIES study. Plos One.

[83] Leong DP, Teo KK, Rangarajan S, Lopez-Jaramillo P, Avezum A Jr, Orlandini A (2015). Prognostic value of grip strength: findings from the Prospective Urban Rural Epidemiology (PURE) study. Lancet.

[84] Lopez-Jaramillo P, Cohen DD, Gómez-Arbeláez D, Bosch J, Dyal L, Yusuf S, for the ORIGIN Trial Investigators (2014). Association of handgrip strength to cardiovascular mortality in pre-diabetic and diabetic patients: A subanalysis of the ORIGIN trial. Int J Cardiol.

[85] Leong DP, Teo KK, Rangarajan S, Kutty VR, Lanas F, Hui C (2016). Reference ranges of handgrip strength from 125,462 healthy adults in 21 countries a prospective urban rural epidemiologic (PURE) study. J Cachexia Sarcopenia Muscle.

[86] Lopez-Lopez J, Lopez-Jaramillo P, Camacho PA, Gomez-Arbelaez D, Cohen DD (2015). The link between fetal programming, inflammation, muscular strength, and blood pressure. Mediators Inflamm.

[87] Barker DJ, Martyn CN (1992). The maternal and fetal origins of cardiovascular disease. J Epidemiol Community Health.

[88] Lopez-Jaramillo P, Gomez-Arbelaez D, Sotomayor-Rubio A, Mantilla-Garcia D, Lopez-Lopez J (2015). Maternal undernutrition and cardiometabolic disease A Latin America Perspective. BMC Medicine.

[89] López-Jaramillo P, Silva S (2008). Y, Rodríguez-Salamanca N, Duran A, Mosquera W, Castillo V Are nutrition-induced epigenetic changes the link between socioeconomic pathology and cardiovascular diseases?. Am J Ther.

[90] Perez M, Gonzales L, Olarte R, Rodriguez NJ, Tabares M, Salazar JP (2011). Nonalcoholic fatty liver disease is associated with insulin resistance in a young Hispanic population. Prev Med.

[91] Gomez-Arbelaez D, Lopez-Jaramillo P (2013). Mechanisms of acute coronary syndromes. N Engl J Med.

[92] Lopez-Jaramillo P, Gomez-Arbelaez D, Lopez-Lopez J, Lopez-Lopez C, Martinez-Ortega J, Gomez-Rodriguez A (2014). The role of leptin-adiponectin ratio in metabolic syndrome and diabetes. Horm Mol Biol Clin Investig.

[93] Lopez-Jaramillo P (2016). The role of adiponectin in cardiometabolic diseases effecs of nutritional interventions. J Nutr.

[94] Lopez-Jaramillo P, Gonzalez-Gomez S, Vanstrahlen-Gonzales L, Zarate-Bernal D, DiStefano K, Camacho-Lopez P (2015). The interaction between epigenetic, muscle, and cardiovascular diseases. Clinical Epigenetics.

[95] Block T, El-Osta A (2017). Epigenetic programming, early life nutrition and the risk of metabolic disease. Atherosclerosis.

