# Supplementary material for: Prediabetes in Colombia: Expert Consensus
Source: Colomb Med (Cali). 2017 Dec 30;48(4):191–203. doi: 10.25100/cm.v43i4.3662 (PMC5896726; doi:10.25100/cm.v43i4.3662)
Supplement: Related texts

# Review article: Prediabetes en Colombia: Consenso de expertos

## Introducción

Convocados por la Federación Diabetológica Colombiana representantes de la Asociación Colombiana de Endocrinología (ACE), de la Asociación Colombiana de Medicina Interna (ACMI), de la Sociedad Colombiana de Cardiología (SCC), de la Fundación Colombiana de Obesidad (FUNCOBES), de la Sociedad Colombiana de Sarcopenia (SCS) y de la Sociedad Latinoamericana de Hipertensión (LASH) nos reunimos en Agosto de 2017 para revisar los conceptos actuales sobre las alteraciones de la glucosa que preceden a la diabetes mellitus tipo 2 (DM2) y que se conocen como prediabetes, el comportamiento epidemiológico a nivel mundial y particularmente en Colombia, las implicaciones en la fisiopatología de las enfermedades cardiovasculares (ECV). Además, analizar la posición de las guías internacionales y nacionales en torno a la definición, los factores de riesgo, el diagnóstico, el tratamiento farmacológico y no farmacológico, las recomendaciones de monitoreo a corto y largo plazo. Este documento contribuirá a mejorar el conocimiento de los médicos generales y especialistas en torno a esta alteración de la glucemia queestá asociada con un mayor riesgo para desarrollar DM2 y ECV ^1^^,^^2^.

## Definición y criterios diagnósticos de la prediabetes

El término prediabetes hace referencia a un estado metabólico intermedio entre una homeostasis normal de la glucosa y la DM2. Se la diagnostica por los niveles de glucosa en sangre venosa, así: glucemia de ayuno alterada (GAA) cuando los valores están entre 100 y 125 mg/dL luego de al menos 8 horas de ayuno, y/o intolerancia a la glucosa (ITG) cuando los valores de glucemia luego de dos hora de la administración de una sobrecarga oral de 75 g de glucosa, se encuentran entre 140 y 199 mg/dL, y/o si los valores de hemoglobina glucosilada (HbA1c) se encuentran entre 5.7% y 6.4% ^1^. 

Otros términos utilizados para referirse a la prediabetes son los de: categoría de glucemia con riesgo aumentado de DM2 y de hiperglucemia intermedia, término propuesto por la Organización Mundial de la Salud (OMS) ^3^.

## Epidemiología de la prediabetes 

De acuerdo a la Federación Internacional de Diabetes (IDF) la prevalencia mundial de prediabetes oscila entre 6% y 14%, y para Colombia la estimación ajustada por edad (20-79 años) es del 8 al 10% ^4^.

La Encuesta Nacional de Situación Nutricional en Colombia (ENSIN) del 2010 ^5^ reportó una incidencia de prediabetes del 5%, sin embargo las determinaciones de glucosa fueron realizadas en sangre capilar, método que subestima las concentraciones de glucemia, lo que podría haber subestimado la prevalencia de prediabetes ^6^. Esta posibilidad se soporta además por el hecho de que la prevalencia de sobrepeso y obesidad, entidades estrechamente asociadas a la prediabetes, son mas altas, así, el 50% de los adultos mayores de 35 años tienen sobrepeso y obesidad. En las personas mayores de 18 años el sobrepeso y la obesidad abdominal aumentaron desde la ENSIN 2005 ^7^ a la ENSIN 2010 ^5^ (Tabla 1). La ENSIN del 2010 reportó una prevalencia de sobrepeso de 17% entre los niños y jóvenes de 10 a 17 años ^5^. El estudio Prospective Urban and Rural Epidemiology (PURE)-Colombia que se desarrolló en 11 departamentos de Colombia y que incluyó 7,500 adultos entre 35 y 70 años, reportó una prevalencia de prediabetes de 11.9% ^8^, mientras un estudio realizado en Barranquilla ^9^ reportó que la prevalencia en adultos de ambos sexos de intolerancia a la glucosa aislada fue del 8% y la de glucemia en ayunas alterada del 11%, mientras que la prevalencia de prediabetes en 439 sujetos con un primer infarto de miocardio fue del 29.6% ^10^, datos que demuestran la importancia de la prediabetes tanto por la alta prevalencia entre la población adulta colombiana, así como por su relación con desenlaces cardiovasculares ^11^.

**Tabla 1:** Prevalencia de obesidad abdominal en Colombia. ENSIN 2005 - 2010

Edad (Años)	Obesidad Abdominal		Obesidad Abdominal	
	Hombres ≥90 cm		Mujeres ≥80 cm	
	2005 (%)	2010 (%)	2005 (%)	2010 (%)
18 a 22	6.6	8.9	18.9	26.7
23 a 27	16.3	22.4	31.5	40.3
28 a 32	28.7	44.0	34.7	54.6
33 a 37	36.1	42.7	52.5	635
38 a 42	44.2	59.0	51.6	71.1
43 a 47	46.8	54.0	65.9	75.1
48 a 52	54.1	73.0	58.8	81.3
53 a 57	56.2	59.4	75.8	85.2
58 a 64	55.2	60.7	77.4	84.9
Total	33.8	39.8	51.4	62.0

Fuente ENSIN 2005-2010

ENSIN: Encuesta Nacional de Situación Nutricional en Colombia 2010

## Conocimiento y sub-diagnóstico de la prediabetes

A pesar de que la prediabetes es reconocida a nivel mundial y aceptada por las principales guías nacionales e internacionales relacionadas con el tema, todavía existe un evidente desconocimiento entre los médicos y el equipo de salud, y como consecuencia una importante falta de evaluación de este estado metabólico ^12^. En Estado Unidos de América (USA) un reciente estudio entre médicos de atención primaria demostró que el 11% de los encuestados contestó adecuadamente todas las preguntas sobre factores de riesgo para prediabetes ^13^. En Colombia ^14^ se aplicó la misma encuesta en 429 médicos que asistieron a dos eventos académicos de medicina interna y diabetes, encontrando que el 9.5% identificaron los doce factores de riesgo propuestos en la encuesta. 

El código para prediabetes existe en la Clasificación Internacional de Enfermedades (CIE-10) vigente en Colombia, y está clasificada como síntomas, signos y hallazgos anormales clínicos y de laboratorio con el Código R730, que corresponde a las anormalidades de la prueba de tolerancia a la glucosa ^15^ (Tabla 2). Este código no ha sido suficientemente difundido entre los médicos colombianos, lo que posiblemente está contribuyendo al sub-diagnóstico de la prediabetes, situación que asumimos a pesar de que los datos disponibles en Colombia con respecto al diagnóstico de prediabetes son escasos, puntuales y de poca calidad metodológica. El estudio de Caracterización de pacientes con Prediabetes en el primer nivel de atención institucional, tuvo como objetivo identificar si existía el diagnóstico de prediabetes en pacientes afiliados a una aseguradora privada de salud conocidas en Colombia como Entidad Promotora de Salud (EPS). Se demostró que a pesar de la existencia de factores asociados a la prediabetes, no se sentó el diagnóstico ni se propuso ningún manejo terapéutico específico ^16^.

**Table 2:** Código CIE 10 que aplica a Prediabetes ^14^

Capítulo	Nombre Capítulo	Código de la CIE-10 a tres caracteres	Descripción de códigos a tres caracteres	Código de la CIE-10 a cuatro caracteres	Descripción de códigos a cuatro caracteres	Inclusión
18	Síntomas, signos y hallazgos anormales clínicos y de laboratorio, no clasiﬁcados en otra parte	R73	Nivel elevado de glucosa en sangre			Excluye: -Diabetes mellitus (E10-E14) -En embarazo, parto y puerperio (O24.-) -Hipoinsulinemia postquirúrgica (E89.1) -Trastornos neonatales (P70.0-P70.2)
18	Síntomas, signos y hallazgos anormales clínicos y de laboratorio, no clasiﬁcados en otra parte	R73	Nivel elevado de glucosa en sangre	R730	Anormalidades en la prueba de tolerancia a la Glucosa	DIABETES: -Lactante Química
						PREDIABETES:-Tolerancia a la glucosa alterada
18	Síntomas, signos y hallazgos anormales clínicos y de laboratorio, no clasiﬁcados en otra parte	R73	Nivel elevado de glucosa en sangre	R739	Hiperglicemia, no especiﬁcada	

## Progresión de Prediabetes a DM2 e impacto en eventos cardiovasculares

La DM2 es una enfermedad progresiva que se inicia con una larga fase asintomática en individuos que presentan diferentes factores de riesgo ^17^, los cuales han sido identificados en estudios poblacionales como el estudio PURE-Global que reportó que la mayor edad, el sexo masculino, la residencia urbana, el bajo nivel educativo, la baja actividad física, los antecedentes familiares de DM2, el aumento del índice de masa corporal (IMC) y el aumento de la relación cintura cadera (C/C), son los factores de riesgo identificados en todos los países participantes, siendo los tres últimos los más importantes ya que aumentan en 3.15, 2.76 y 3.63 veces respectivamente, el riesgo de presentar DM2 ^18^. Estos factores de riesgo son universales, sin embargo este estudio demostró que la prevalencia de DM2 fue 6 veces más alta en los individuos con IMC menor a 21 residentes en países de bajos ingresos, en relación con aquellos residentes en países de altos ingresos económicos. Interesantemente esta mayor sensibilidad de los individuos de países de bajos ingresos a presentar mayor prevalencia de DM2 a menores niveles de IMC, también se observó en las tasas de progresión de prediabetes a DM2, así, mientras en USA el *Diabetes Prevention Program* (DPP) demostró una progresión anual del 11% ^19^, el *Diabetes Prevention Study* (DPS) en Finlandia del 6% ^20^, el *Indian Diabetes Pevention Program* (IDPP) mostró una progresión anual del 18% ^21^, y en Colombia ^22^ se reportó una progresión anual del 9%. 

Proyectando el número de la población de Colombia para el 2018 ^23^ , habrá para ese año 3.2 millones de pacientes prediabéticos adultos, de los cuales 2.1 millones no estarán diagnosticados ni tratados, es decir dos de cada tres prediabéticos colombianos esta desprotegido del riesgo de volverse diabético o presentar eventos cardio-vasculares ^11^ (Fig. 1).

**Figura 1:** Estimado del número de sujetos con Prediabetes en Colombia 2018

La importancia de la identificación de individuos con prediabetes radica en la posibilidad de que su manejo temprano detenga el crecimiento en la incidencia de la DM2 que está ocurriendo actualmente ^24^ , especialmente en los países de ingresos medios y bajos ^4^ . 

Datos recientes de la IDF ^4^ demuestran que el número de personas con DM2 se ha elevado de 135 millones en 1995 a 415 millones en 2015, siendo que la prevalencia se ha elevado más rápidamente en países de ingresos moderados a bajos, y para el año 2040 se esperan 642 millones de pacientes diabéticos, es decir un incremento del 64% de la población diabética.

En el año 2015 5 millones de muertes se atribuyeron a DM2, el 43% de esta mortalidad se presentó en pacientes por debajo de los 50 años ^4^.

Además, la DM2 es la mayor causa de ceguera, falla renal, infarto de miocárdico, accidente cerebro-vascular y amputación no traumática de miembros inferiores, complicaciones que en parte son el resultado de la falta de un diagnóstico temprano y de una intervención oportuna para controlar la enfermedad desde el estado de prediabetes ^25^^,^^26^. 

La prediabetes además de ser un factor de riesgo importante para el desarrollo de DM2, lo es también para ECV, Se ha demostrado que en el estado prediabético ya existe daño vascular, cuya gravedad depende del tiempo de evolución de la hiperglucemia, ya que la glucosa crónicamente elevada ocasiona daño pan-vascular: macro y micro-angiopatía, por dos mecanismos inherentes a la enfermedad aterosclerótica: oxidación e inflamación vascular ^27^^-^^31^. En la prediabetes ya coexisten estos fenómenos, y cuando se diagnostica la DM2, años más tarde, el daño vascular ya se ha magnificado por el mecanismo de Memoria Metabólica (MM), en el cual hay oxidación de la cadena de citocromos y la mitocondria transforma su producción de ATP en especies reactivas de oxígeno, las cuales llevan a apoptosis de las células endoteliales y un daño irreversible de la pared vascular ^28^^-^^31^. De ahí la importancia de controlar la hiperglucemia desde el inicio mismo de la prediabetes para evitar el daño vascular ^32^ y su perpetuación a través de la MM ^29^. 

El tiempo de espera para que la hiperglucemia alcance los niveles de corte actualmente aceptados para hacer el diagnóstico de DM2 e intervenir, determina que el daño vascular avance y que se vuelva irreversible, como está demostrado por la mayoría de ensayos clínicos en pacientes con DM2 con más de cuatro años de evolución en los cuales las terapias intensificadas o con nuevos hipoglucemiantes, a pesar de alcanzar los niveles recomendados de HbA1C, no han tenido efecto en disminuir los eventos CV ^33^^,^^34^, mientras que el estudio UKPDS en pacientes con un reciente diagnóstico de DM2, demostró una reducción de los eventos CV ^35^^,^^36^. 

Es conocido que desde el punto de vista fisiológico existen células que no poseen la capacidad de regular el transporte de glucosa en presencia de hiperglucemia, de ahí que las complicaciones de la DM2 se presenten en los tres tipos de células que no se adaptan a esta circunstancia: retina, mesangio y neuronas ^31^. El daño en estas células comienza cuando los niveles de glucemia en ayunas todavía son normales, pero ya existen picos hiperglucémicos post-prandiales amplios ^37^, lo que ha llevado a la propuesta de que el mecanismo crucial del daño vascular se origina por la resistencia a la insulina, que se caracteriza por una estrecha conexión entre hiperglucemia, aumento de LDL densa y pequeña, disfunción del endotelio vascular, alteraciones morfológicas de la pared vascular y de la coagulación, todo lo cual explica por qué el paciente con hiperglucemia crónica presenta desenlaces aterotrombóticos más frecuentes a través de que todos estos factores conducen al gran problema vascular del enfermo diabético que es el Síndrome de Envejecimiento Vascular Precoz (EVA), que comienza desde el inicio mismo de la resistencia a la insulina y que se manifiesta como rigidez arterial, aumento en la velocidad de onda de pulso, onda de rebote aumentada con el consiguiente índice de aumento, incremento de las presiones centrales y de la presión de pulso central, alteraciones todas que se manifiestan mucho antes que aparezca la hipertensión arterial medida por la tradicional presión arterial braquial ^38^^,^^39^. Por lo tanto, es importante detectar el proceso tempranamente para evitar las complicaciones cerebro-cardio-reno-angio-vasculares. No obstante, la detección y el tratamiento de la prediabetes no es una conducta común en nuestro sistema de salud. La dupla Cardio-Metabólica y Diabetes-Hipertensión, son bombas de tiempo y detonantes para desenlaces CV precoces, situación que se agrava por el mal manejo o el no manejo integral de todos los factores de riesgo, conducta necesaria para preservar o restaurar la adecuada funcionalidad de la pared vascular. En concluisón la falta de diagnóstico y tratamiento de la prediabetes son factores importantes para la prevención ^40^.

## Identificación de sujetos en riesgo de Prediabetes y DM2

Las escalas de riesgo son herramientas de tipo cuestionario, sencillas y útiles, que permiten un modelo más costo-efectivo del tamizaje de diferentes enfermedades. Para la identificación de sujetos en riesgo de Prediabetes y DM2, el *Finnish Diabetes Risk Score* (FINDRIS) ha demostrado ser simple, rápido, económico, no invasivo y confiable y ha sido evaluado en varios países de diferentes ingresos y culturas. Han arrojado diferentes puntos de corte asociados al riesgo, así como también diferencias en la sensibilidad y especificidad ^41^^,^^42^.

El FINDRIS incluye los factores de riesgo para DM2 identificados en varias poblaciones, y que dieron lugar a un cuestionario de ocho preguntas: edad, IMC, actividad física, ingesta de frutas y hortalizas, tratamiento médico de hipertensión, historia de hiperglucemia y la historia familiar de diabetes. Es un cuestionario que no requiere pruebas de laboratorio (Tabla 3).

El cuestionario da una puntuación a cada uno de los factores de riesgo para DM2, de modo que la suma total de los puntos clasifica como bajo, moderado, alto y muy alto, a las personas según su riesgo de desarrollar DM2 en los próximos 10 años .Para la población finlandesa los puntos de corte del cuestionario fueron propuestos así ^41^:

1. Puntuación de riesgo de 0 a 14 puntos indica un riesgo bajo o moderado de diabetes (1-17% de probabilidades de DM2 en 10 años).
2. Puntuación de riesgo de 15-20 puntos indica un alto riesgo de diabetes (33% de probabilidades de DM2 en 10 años).
3. Puntuación de riesgo >20 puntos indica un riesgo muy alto de diabetes (50% de probabilidades de DM2 en 10 años).

**Table 3:** Escala de Riesgo FINDRISC validada en Colombia. Prueba FINDRISC para tamizaje de Diabetes u otras anormalidades de la regulación de la glucosa. Adaptado de la referencia 54

Nombre	Telefono
1. Edad. años cumplidos	⎕Less than 45 years (0 p.)
	⎕ Menos de 45 años (0 p.)
	⎕ 45-54 años (2 p.)
	⎕ 55-64 años (3 p.)
	⎕ Más de 64 años (4 p.)
2. Índice de masa corporal:	Peso. Kg
	Talla. m
IMC:	⎕ Menor de 25 kg/m² (0 p.)
	⎕ Entre 25-30 kg/m² (1 p.)
	⎕ Mayor de 30 kg/m² (3 p.)
kg/m² *(Se calcula dividiendo el peso en kilos) por la talla en metros elevada al cuadrado. Ej.: Peso 70 kg/Talla 1.70 m al cuadrado = 70/2.89 = 24.2 kg/m²)*	
3. Perímetro de cintura. Cm	*Pasar el metro en medio del reborde costal y la cresta ilíaca de ambos lados y totalmente horizontal (aunque no pase por el ombligo). La persona debe respirar normalmente un par de veces y se toma la medida menor (cuando exhale el aire)*
Hombres	⎕ Menos de 94 cm (0 p.)
	⎕ Igual o mayor a 94 cm (4 p.)
Mujeres	⎕ Menos de 90 cm (0 p.)
	⎕ Igual o mayor a 90 cm (4 p.)
4. ¿Realiza habitualmente (a diario) al menos 30 minutos de actividad física, en el trabajo y/o en el tiempo libre?:	⎕ Si (0 p.)
	⎕ No (2 p.)
5. ¿Con qué frecuencia come verduras o frutas?:	⎕ Todos los días (0 p.)
	⎕ No todos los días (1 p.)
6. ¿Toma medicación para la hipertensión regularmente?:	⎕ No (0 p.)
	⎕ Yes (2 p.)
7. ¿Le han encontrado alguna vez valores de glucosa alta?	*(Ej. en un control médico, durante una enfermedad, durante el embarazo)*
	⎕ No (0 p.)
	⎕ Si (5 p.)
8. ¿Se le ha diagnosticado diabetes (tipo 1 o tipo 2) a alguno de sus familiares?	⎕ Sí: padres, hermanos o hijos (5 p.)
	⎕ Sí: abuelos, tíos, primos hermanos (3 p.)
	⎕ Otros parientes o ninguno (0 p.)
*(Nota: La diabetes "por edad" o "ya de viejo" también cuenta)*	
Escala de riesgo total.	
Sume los puntos de las respuestas contestadas, para conocer su riesgo.	
Puntaje Total (máximo 26 p)	
Interpretación: Si la puntuación del Test es igual mayor a 12, usted tiene alta probabilidad de tener Diabetes u otra anormalidad de la regulación de la glucosa.		

Sin embargo, la recomendación de quienes desarrollaron este cuestionario ^41^^,^^42^ fue la de validar el instrumento en cada país, por lo que en Colombia ^43^ realizamos un estudio poblacional para evaluar el cuestionario FINDRIS y establecer los puntajes a los cuales en nuestra población se incremente el riego de DM2. Se demostró que FINDRIS es una herramienta útil de tamizaje para identificar sujetos con DM2 no conocida y para predecir la incidencia de DM2 entre los prediabéticos. El punto de corte para predecir DM2 en prediabéticos es de 13 en hombres y 16 en mujeres ^43^. Otro grupo ^9^ también implementó un estudio con el fin de desarrollar un modelo de calificación de riesgo para la población colombiana denominándole ColDRIS y para evaluar la exactitud del ColDRIS para detectar DM2 no diagnosticada. El cuestionario se adaptó y validó en la población colombiana con el único cambio en los puntos de corte de la circunferencia abdominal, documentándose una sensibilidad de 74% y una especificidad de 60%, para detectar individuos en riesgo de DM2 con un puntaje igual o mayor a 12. Estos valores sugieren que es baja la cantidad de falsos negativos y, según la información disponible en la literatura, estos pacientes no presentarán complicaciones en los siguientes tres años, momento en el cual se recomienda una nueva tamización. El ColDRIS acortado considera cuatro variables: edad, perímetro de cintura, uso de medicamentos para control de la presión arterial e historia personal de diabetes, y a diferencia del FINDRIS no considera dieta, sedentarismo, historia de glucosa e IMC.

Los dos estudios realizados en Colombia demuestran que el FINDRIS puede ser utilizado como una prueba simple, segura y sin costo para identificar las personas con alto riesgo de desarrollar DM2, por lo que el Consenso recomienda que se hagan los esfuerzos necesarios para lograr introducir la encuesta FINDRIS de manera universal en los niveles de atención primaria, que permita definir el nivel de riesgo del paciente antes de solicitar las pruebas de laboratorio, entre las cuales la determinación de la HbA1C como prueba diagnóstica para prediabetes o DM2 es controversial ya que existen datos controversiales sobre su utilidad para agregar sensibilidad y/o especificidad para la identificación de individuos con prediabetes o DM2, y se ha reportado que no es una herramienta de diagnóstico mejor que la glucemia en ayunas o la POTG ^44^^-^^50^. En Colombia un reciente trabajo sugiere que esta prueba debe ser utilizada únicamente para el seguimiento de pacientes en los que se debe evaluar el efecto de los tratamientos implementados, de tal manera de reducir los costos para el sistema de salud ^44^.

Para el Consenso la prueba diagnóstica más apropiada para el diagnóstico de prediabetes es la medición de una glucemia plasmática en ayunas y en caso de resultados dudosos, corroborar con la PTOG, pruebas que deben ser solicitados si, como se propone en la Guía Colombiana de Práctica Clínica para el diagnóstico, tratamiento y seguimiento de la DM2 en la población mayor de 18 años, el puntaje de FINDRIS en adultos es igual o mayor a 12 (Recomendación fuerte a favor, Calidad de la evidencia: moderada). En la Figura 2 se propone un algoritmo para la evaluación y manejo del riesgo de alteraciones de la glucemia.

**Figura 2:** Supervivencia de pacientes colombianos luego de un primer infarto de miocardio por estado de glucemia evaluada al momento del alta hospitalaria

## ¿Qué dicen las guías acerca de la prediabetes?

La *Guía de Práctica Clínica de Colombia para el diagnóstico, tratamiento y seguimiento de la diabetes mellitus tipo 2 en la población mayor de 18 años / 2015 Guía No. GPC-2015-51*, reconoce el término de riesgo aumentado de diabetes o prediabetes, señalando que las personas con puntaje igual o mayor a 12 en la escala Findrisk que no cumplen criterios diagnósticos de DM2, se les recomienda establecer la presencia de categorías de riesgo aumentado de diabetes para incluirlas en programas de prevención de DM2 ^51^. Las características con riesgo aumentado de diabetes son glucemia de ayuna alterada (GAA) entre 100 y 125 mg/dL y/o intolerancia a la glucosa (ITG) con glucemia a las 2 horas post carga de 75 g de glucosa oral entre 140 y 199 mg/dL.

Las *Guías de la Asociación Latinoamericanas de Diabetes (ALAD) sobre el diagnóstico, control y tratamiento de la DM2 con Medicina Basada en la Evidencia*, también reconoce el término y explica los criterios para el diagnóstico de DM2 y de los trastornos de la regulación de la glucosa ^52^^,^^53^. Esta guía mantiene los valores anteriormente expuesto para GAA e ITG, pero también considera valores de HbA1c entre 5.7 y 6.4% para diagnosticar Prediabetes, al igual que las guías nuevas de la ADA 2017 ^1^.

El *Consenso de la Asociación Americana de Endocrinología y el Colegio Americano de Endocrinología* publicaron un algoritmo para tratar al paciente con Prediabetes, iniciando siempre con cambios en el estilo de vida. Los parámetros más importantes a considerar son la medición de los factores de riesgo CV, las medidas para normalizar el peso y el tratamiento de la hiperglucemia para normalizar la GAA o la ITG ^54^. 

Las *Guías de la American Diabetes Association (ADA) 2017*, establecieron los criterios para evaluar la presencia de DM2 y Prediabetes en adultos asintomáticos y establecieron los factores de riesgo como se detalla en la Tabla 3. ^1^. 

Las *Guías de la Sociedad Europea de Cardiología en colaboración con la Asociación Europea para el Estudio de la Diabetes*, publicaron unas guías en torno a la DM2, Prediabetes y Enfermedades Cardiovasculares, donde establecieron recomendaciones para el diagnóstico oportuno de Diabetes o Prediabetes en pacientes con Enfermedad CV. Ellos recomiendan tamizar para DM2 a pacientes con Enfermedad Cardiovascular (ECV) basándose primordialmente en la HbA1c y en la glucemia en ayunas. Recomiendan realizar una prueba oral de tolerancia a a glucosa (POTG) cuando la HbA1c o la glucemia en ayunas no fueran concluyentes ^55^.

La *Organización Mundial de la Salud (OMS),* en su reporte del año 2006 reconoce la hiperglucemia intermedia e indica que se puede diagnosticar con la prueba de tolerancia a la glucosa alterada (entre 140 y 200 mg/dL) y/o con glucemia en ayunas alterada (entre 110 y 125 mg/dL) ^3^.

## Manejo de la prediabetes para la prevención de DM2 y eventos cardiovasculares

Varios estudios y meta-análisis han demostrado que el riesgo de ECV aumenta con valores de glucemia en el rango de prediabetes y que el aumento de la HbA1C en valores de prediabetes no solo que se asocia con mayor progresión a DM2 sino con mayor riesgo de ECV ^56^^-^^61^.

Un estudio en Colombia demostró que la hiperglucemia se asoció a mayor número de desenlaces adversos en 439 individuos que sobrevivieron a un primer infarto agudo de miocardio (IAM), así, el 70% de los individuos que tuvieron un IAM tenían hiperglucemia, pero solamente el 20% eran diabéticos conocidos con larga historia de evolución, en el 20% de los pacientes el diagnóstico de DM2 se hizo cuando se presentó el infarto y el 30% de los pacientes eran prediabéticos ^62^. Durante el seguimiento por un período de tres años, la supervivencia de los pacientes con glucemia normal fue mayor que la de los pacientes con alteraciones de la glucosa independientemente de si fueron diabéticos de larga evolución, de reciente diagnóstico o prediabéticos. Como se muestra en la Figura 3, en los primeros meses post infarto de miocardio la mortalidad en los pacientes con DM2 y mayor tiempo de duración de la hiperglucemia fueron los que presentaron menores tasas de supervivencia, a pesar de recibir la más intensificada terapia farmacológica. Al final del seguimiento de tres años, las tasas de supervivencia de los tres grupos con alteración de la glucosa fueron similares y menores que la de los normo- glucémicos, lo que demuestra que la hiperglucemia es un factor que aumenta el riesgo de mortalidad, independientemente de si los niveles de glucosa son los que determinan la denominación de prediabetes o diabetes. Sin embargo, mientras los pacientes con DM2 recibieron tratamiento hipoglucemiante de acuerdo a las guías, los prediabeticos, de acuerdo a esas mismas guías, no recibieron metformina que es el tratamiento farmacológico de primera línea para el manejo de la hiperglucemia, por lo que no tenemos evidencia en estos pacientes colombianos de si el manejo con metformina en nuestros pacientes prediabéticos podría haber aumentado la tasa de supervivencia. 

**Figura 3:** Algoritmo propuesto para la evaluación del riesgo y manejo de alteraciones de la glucemia

Los datos del UKPDS en pacientes con DM2 de reciente inicio que recibieron metformina tuvieron menos eventos CVs ^35^^,^^36^, lo que sugiere que nuestros pacientes prediabéticos podrían haberse beneficiado del tratamiento con metformina no solo para disminuir la progresión de prediabetes a DM2, sino también para reducir eventos CV. Estos datos han servido para cuestionar la utilidad en países de medianos y bajos ingresos de los puntos de corte de glucemia actualmente utilizados para establecer el diagnóstico de DM2, los cuales vienen de un estudio finlandés que evaluó la asociación de glucemia con retinopatía y no con desenlaces CV ^63^. Existe un punto de no retorno durante el curso de la neuropatía periférica, la cardiomiopatía, y la nefropatía diabética, cuando la hiperglucemia crónica ocasiona alteraciones funcionales y alteraciones estructurales, como fue demostrado elegantemente por un grupo en Londres ^64^^,^^65^, quienes observaron en un modelo experimental de diabetes ocasionada por la administración de estreptozotocina, sustancia que destruye las células beta pancreáticas en ratas, que si el control de la hiperglucemia con insulina se inicia inmediatamente y hasta cuatro semanas luego de inducida la diabetes, se previene de que ocurran las lesiones estructurales en los nervios nitrérgicos, pero si el tratamiento se inicia luego de ocho semanas de hiperglucemia, la administración de insulina normaliza la glucemia, pero ya no revierte los cambios estructurales que se manifiestan por las complicaciones micro y macro vasculares de la diabetes. Por lo tanto, como propuesto desde hace varios años ^66^ para tener resultados positivos de prevención cardiovascular primaria en pacientes con hiperglucemia es necesario actuar controlando desde que se detecta la prediabetes y los factores de riesgo asociados, como el sobrepeso y la obesidad, la hipertensión y la dislipidemia, tanto con cambios terapéuticos en los hábitos de vida, como también con medicamentos como metformina, estatinas e inhibidores del sistema renina-angiotensina-aldosterona (SRAA). Recientemente el estudio HOPE 3, demostró que la administración de estos dos últimos medicamentos redujo en 40% el riesgo relativo de ECV en pacientes de riesgo intermedio ^67^^-^^69^. Cuando el paciente ya es diabético de larga duración y el daño ocasionado por la hiperglucemia en el sistema vascular y en otros tejidos está presente, la mayoría de los estudios clínicos han demostrado el fracaso de prevenir desenlaces por las complicaciones macrovasculares, ya sea con tratamiento intensificado o por la utilización de nuevos hipoglucemiantes ^33^^,^^34^, momento en el cual, como demostrado por el estudio STENO lo más importante es el control riguroso de la presión arterial y los lípidos, por sobre intentar tardíamente controlar estrictamente los niveles de glucemia ^66^. El punto clave para la prevención de ECV asociadas a la hiperglucemia no es cuánto se logre bajar la HbA1c, sino el momento en el cual se inicia el control de la hiperglucemia, concepto que también se relaciona con la fisiopatología de las complicaciones vasculares de la diabetes, ya que existe evidencia del importante papel que juegan los productos terminales avanzados de glucación (*advanced glycation endproducts* - AGEs-) en el desarrollo de estas complicaciones ^70^. Mientras mas tiempo se mantiene la hiperglucemia mayor es la formación de estos productos, y además, comienzan a glucosilarse proteínas estructurales de las membranas celulares, proceso que es de naturaleza irreversible. La metformina si utilizada a tiempo tiene la capacidad de influenciar los cambios mediados por los AGEs en la estructura y la función de varios tejidos y sistemas. En un modelo canino de diabetes se demostró que cuatro meses de tratamiento con metformina redujo en forma importante tanto la rigidez miocárdica como el contenido de colágeno glucosilado, mostrando que con la metformina se puede controlar los cambios deletéreos asociados con la glucación de las proteínas *in vivo* ^70^. 

Con estos antecedente, el Consenso sugiere que los laboratorios que comercializan metformina realicen las gestiones pertinente para obtener la aprobación por parte del Instituto Nacional de Vigilancia de Medicamentos y Alimentos (INVIMA), la agencia reguladora de medicamentos en Colombia, de la indicación de metformina como tratamiento coadyuvante a los cambios terapéuticos en los hábitos de vida para el manejo de la prediabetes, como ya se ha realizado en varios países desarrollados como el Reino Unido y en algunos Latinoamericanos como Perú y México, y que una vez aprobada la indicación se promocione la indicación de metformina para el manejo de prediabetes a través de programas de educación médica continuada. 

Recalcamos que el ejercicio, la dieta saludable y la pérdida de peso son medidas importantes para el manejo de la prediabetes, pero que además si se dispone de una herramienta farmacológica que coadyuva a disminuir la glucemia y evitar tanto la progresión a DM2, como las complicaciones macro y microvasculares, pero cuyo uso en Colombia se ha visto limitado por la falta de conocimiento de los médicos de la importancia del diagnóstico y manejo de la prediabetes como también por la no aprobación por parte del ente regulatorio de la indicación de metformina para el tratamiento coadyuvante de la prediabetes, medida que debería implementarse de acuerdo a las recomendaciones de varias guías como que revisamos anteriormente.

El como prevenir la progresión de prediabetes a DM2 es un tópico importante tanto para las organizaciones académicas del mundo entero como para las entidades encargadas de la salud pública en nuestro país ^71^. Varios estudios sobre intervención en el estilo de vida y/o metformina han demostrado una reducción sostenida en la tasa de progresión de prediabetes a DM2, así, el Diabetes Prevention Study (DPS), trabajo finlandés ^20^ realizado en una población de alto riesgo para diabetes, con historia de familiares directos con DM2, sobrepeso (IMC ≥25 kg/m^2)^, con edades entre 40 y 65 años e intolerancia a la glucosa, incluyó 523 personas que fueron aleatorizadas a recibir intervención con cambios en el estilo de vida (dieta y ejercicio) con unapérdida de peso del 5% o más, o a grupo control. Se demostró que la incidencia acumulada de diabetes luego de 4 años fue de 11% en el grupo de intervención y de 23% en el grupo control. Durante el estudio el riesgo de diabetes se redujo en 58% (*p* <0.001) en el grupo de intervención. La reducción de la incidencia de diabetes estuvo directamente asociada con los cambios en el estilo de vida. El estudio concluyó que la DM2 puede prevenirse con cambios en el estilo de vida en sujetos de alto riesgo. El estudio Diabetes Prevention Program (DPP) realizado en USA ^19^ fue un ensayo clínico, aleatorizado, multicéntrico, en pacientes mayores de 25 años, con IMC mayor a 24, glucemia en ayunas alterada e intolerancia a la glucosa, que tuvo un seguimiento promedio de 2.8 años. Fueron aleatorizados 3,234 pacientes (68% mujeres) para recibir alguna de las tres intervenciones: programa intensivo para modificar el estilo de vida (n= 1,078), recomendaciones habituales sobre estilo de vida más metformina 850 mg dos veces por día (n=1,073) y recomendaciones habituales sobre estilo de vida más placebo (n= 1,082). La progresión a DM2 fue de 4.8 caso/100 personas-año en el grupo con intervención intensiva en cambios del estilo de vida, de 7.8 en el grupo de metformina y de 11.0 en el grupo placebo. La intervención intensiva en el estilo de vida redujo la incidencia en 58% y la metformina en un 31%, cuando se compararon con el grupo placebo. El estudio demostró que un programa intensivo de modificación del estilo de vida y el tratamiento con metformina son efectivos para prevenir o retrasar la incidencia de DM2 en una población de alto riesgo. Los mejores resultados de la intervención de estilo de vida sobre la metformina fueron más efectivos en las personas mayores de 60 años y en las personas con un índice de masa corporal más bajo, mientras la metformina fue mejor en las personas más jóvenes y en aquellas con un mayor índice de masa corporal (≥35). Esta cohorte tuvo un seguimiento posterior de 10 años para evaluar la incidencia de DM2 y la evolución de la pérdida de peso ^72^ en 2,766 de los pacientes incluidos en el estudio original, 910 del grupo de cambios intensivos en el estilo de vida, 924 del grupo metformina y 932 del grupo placebo. Al final de los 10 años de seguimiento la incidencia de DM2 se redujo en 34% en el grupo de cambios intensivos en el estilo de vida, y en 18% en el grupo de metformina, comparado con el grupo control. El análisis específico de la fase de seguimiento luego de terminado el estudio original demostró una progresión de prediabetes a DM2 de 5.9 % personas-años para la intervención intensiva en cambios en el estilo de vida, de 4.9 % personas-años para el grupo metformina, y de 5.6 % personas-años para el grupo placebo, por lo tanto, la metformina disminuyó la progresión a nuevos casos de DM2 en un 13% en relación al grupo placebo, mientras que en el grupo de cambios intensivos en el estilo de vida la progresión a DM2 fue 5% más alta que en el grupo control. Los mejores resultados obtenidos con metformina en el seguimiento a largo plazo en relación con el grupo de cambios intensivos en el estilo de vida, se explican por la progresiva y gradual ganancia de peso que se observó en este grupo, confirmando la dificultad práctica de mantener la pérdida de peso y el control de la hiperglucemia con cambios en el estilo de vida a largo plazo. Este estudio continuo para el seguimiento para evaluar no solo la progresión a DM2, sino también los efectos a largo plazo de la intervención en cambios en el estilo de vida y de la metformina sobre el desarrollo de complicaciones microvasculares durante 15 años de seguimiento, fase conocida como DPP Outcomes Study (DPPOS) ^73^. Luego de 15 años de la intervención, el promedio anual de incidencia de diabetes fue 7.0%, 5.7%, y 5.2% en los grupos placebo, metformina y de intervención en el estilo de vida, respectivamente. La incidencia de diabetes fue 27% menor en el grupo con intervención en el estilo de vida y 18% menor en el grupo con metformina en relación con el grupo placebo. La incidencia acumulada de diabetes a los 15 años fue de 55% en el grupo cambio en el estilo de vida, de 56% en el grupo metformina, y de 62% en el grupo control. Al final del estudio la prevalencia de complicaciones microvasculares fue mayor en el grupo placebo, pero no hubo diferencias significativas entre los dos grupos de tratamiento. Además de la menor tasa de progresión a DM2 en la cohorte DPPOS, se reportaron otros beneficios de la intervención en el estilo de vida y de la metformina, como la reducción de los factores de riesgo para ECV, reducción en la prevalencia de síntomas del tracto urinario inferior asociados con obesidad y diabetes, y mejoría en la calidad de vida.

Cuando se analizó la relación costo-efectividad a los 10 años de implementadas las intervenciones de cambios en el estilo de vida o metformina para la prevención de diabetes utilizando un análisis por intención de tratar de los estudios DPP/DPPOS ^74^, se concluyó que los cambios en el estilo de vida fueron costo-efectivos al igual que la metformina en comparación con el grupo control y que la inversión en las intervenciones de cambio en el estilo de vida y metformina para evitar la progresión de prediabetes a DM2 en adultos de alto riesgo provee una buena relación costo efectividad. Así, se estimó que l*os* costos médicos directos, acumulativos, per cápita, sin descuento de las intervenciones, tal como se implementaron en el DPP, fueron mayores para el estilo de vida (4,572 dólares americanos) que para metformina (2,281 dólares americanos) o para el grupo control ($769 dólares americanos). Cuando se consideró un descuento para el grupo de cambios en el estilo de vida, basado en que durante el periodo de tres años del DPP los pacientes recibían entrenamiento en sesiones de 10 participantes, el monto per cápita fue de 2,995 dólares americanos, lo que demuestra, que al menos en USA, para disminuir la progresión de prediabetes a DM2 es más costoso inducir cambios en el estilo de vida que administrar metformina.

El DPP dio lugar a un protocolo similar implementado en la India conocido como el Indian Diabetes Prevention Program (IDPP) ^21^ que incluyó 402 prediabéticos, estudio que confirmó que los cambios en el estilo de vida y la metformina previenen la DM2 en similar proporción, pero sin efectos sinérgicos cuando implementados conjuntamente. Sin embargo hay que destacar que los pacientes del IDPP en promedio fueron 5 años más jóvenes, tuvieron un perímetro abdominal promedio 10 cm menor y un IMC 8 puntos menor que los norteamericanos del DPP, y además que los promedios de glucemia en ayunas y post carga fueron ligeramente menores (Tabla 8). La comparación de estos dos estudios ^75^ demuestra claramente la mayor sensibilidad de las poblaciones de países de medianos y bajos ingresos para desarrollar DM2 a menores niveles de adiposidad visceral e IMC, como confirmado recientemente por el estudio PURE ^18^, y también la mejor respuesta a la intervención con metformina; así, la tasa de progresión de prediabetes a DM2 fue de 18.3% por año en los indios-asiáticos, mientras en los norteamericanos fue de 11.0% y en los finlandeses del DPS de apenas 6.0%. Además, la metformina a una dosis de 500 mg/día fue más efectiva para reducir la progresión de prediabetes a DM2 en los indios-asiáticos, con una reducción anual de 14.5%, mientras en los norteamericanos en quienes se utilizó 1,700 mg/día la reducción anual fue de 7.2%, lo que significa que en la población de un país de ingresos bajos como la India, el número necesario a ser tratado para prevenir un caso de DM2 es de 6.9, mientras en USA sube a 13.9. 

**Table 4:** Comparación de las características de los individuos incluidos en tres estudios para la prevención de DM2

	DPP			IDPP			DPS	
Características	Placebo (n: 1,082)	Metformina (n: 1,073)	Cambio estilo de vida (n: 1,079)	Control (n: 136)	Metformina (n:133)	Cambio estilo de vida, (n: 133)	Control (n: 257)	Cambio estilo de vida (n: 265)
Género								
Hombre	335	363	345	104	104	107	81	91
Mujer	747	710	734	32	29	26	176	174
Edad (años)	50.3 ± 10.4	50.9 ± 10.3	50.6 ± 11.3	45.2 ± 5.7	45.9 ± 5.9	46.1 ± 5.7	55.0 ± 7.0	55±7
IMC (Kg/m2)	34.2 ± 6.7	33.9 ± 6.6	33.9 ± 6.8	26.3 ± 3.7	25.6 ± 3.7	25.7 ± 3.3	31.0 ± 4.5	31.3±46
Circunferencia Abdominal (cm)	105.2 ±14.3	104.9 ± 14.4	105.1 ± 14.8	90.8 ± 7.5	89.7 ± 9.5	89.0 ± 7.9	100.5 ± 10.9	102.0 ± 11.0
Glucosa Plasmática (mg/dL)								
En ayunas	106.1 ± 8.4	106.5 ± 8.5	106.3 ± 8.1	99.1 ± 14.4	97.3 ± 14.4	97.3 ± 12.6	110.0 ± 13.0	109.0 ± 14.0
Prueba tolerancia oral a la glucosa	164.5 ± 17.1	165.1 ± 17.2	164.4 ± 16.8	155.0 ± 12.6	153.2 ± 12.6	153.2 ± 12.6	159.0 ± 26.0	159.0 ± 27.0

Media ±Desviación estandard

IMC: Indice de masa corporal

Adaptado de referencia 75

Una reciente y extensa revisión ^76^ sobre el uso de la metformina en Prediabetes y en Prevención de Diabetes, concluyó que existen suficientes evidencias que soporta el uso terapéutico de la metformina para la prevención de la diabetes y menciona que las intervenciones en el estilo de vida son difíciles para mantener por parte de los pacientes; el peso perdido tiende a ser ganado nuevamente y con mayor intensidad con el transcurso del tiempo, mientras que la metformina es eficaz y segura para demorar o prevenir el inicio de la DM2. Además, décadas de uso clínico de la metformina en pacientes con DM2 han demostrado que es generalmente bien tolerada y segura***.***

## Particularidades en Colombia del aumento de prediabetes y DM2

El análisis de los estudios DPS, DPP e IDPP ^75^, más las observaciones epidemiológicas y clínicas realizadas en nuestro medio del crecimiento imparable de la adiposidad visceral y de la DM2 ^77^^,^^78^ llevaron a proponer que a través de mecanismos epigenéticos la programación fetal en madres gestantes de estratos sociales bajos, las que no tienen una adecuada ingesta proteica, sus productos presentan retardo de crecimiento intrauterino y bajo peso al nacer, los cuales en el futuro, y de frente a la invasión de la dieta con hidratos de carbono ultra procesados, tienen mayor sensibilidad para desarrollar resistencia a la insulina, inflamación de bajo grado y DM2 a menores niveles de adiposidad visceral, y por la menor cantidad de masa y fuerza muscular producto del deficiente aporte nutricional recibido de una madre mal nutrida ^79^^,^^80^. En soporte de esta propuesta hemos demostrado en niños de Bucaramanga una mayor concentración de Proteína C Reactiva ultra sensible (PCR), un marcador de inflamaciónón de bajo grado, que la que se observa en niños norteamericanos y europeos de la misma edad y del mismo IMC ^81^, y recientemente confirmamos en niños colombianos con bajo peso al nacer para la edad gestacional y que fueron evaluados a los 10 años, que los que presentaron el cuartil más alto de IMC son los que tuvieron las mayores concentraciones de PCR, de índice HOMA de resistencia a la insulina y mayor cantidad de masa grasa, pero menor fuerza prensil y un mayor índice de riesgo metabólico ^82^. La fuerza prensil es un conocido factor de riesgo para muerte cardiovascular y de cualquier causa en los adultos del estudio PURE ^83^ y en los pacientes con Prediabetes y DM2 del estudio ORIGEN ^84^, evidenciándose claras diferencias en la fuerza prensil entre las poblaciones de los países de bajos y medianos ingresos, que son los mas débiles, en relación con los países de altos ingresos que son los más fuertes ^85^. En verdad hasta ahora se comienza a destacar el importante papel que tiene la masa y la fuerza muscular en la regulación de la inflamación sistémica y de la sensibilidad a la insulina ^86^, dada la conversación cruzada que mantienen estos dos grandes tejidos del organismo para mantener un adecuado balance metabólico y cuya alteración se inicia desde la vida intrauterina, como lo propusiera Baker hace varios años , destacando la importancia de la programación fetal dependiente de la nutrición materna ^87^. En términos prácticos estos resultados demuestran la necesidad de que el paciente prediabético para prevenir la progresión a DM2 tenga un programa de acondicionamiento físico que incluya ejercicios de fuerza-resistencia, además de los cardio-respiratorios [88], especialmente en poblaciones de países de bajos ingresos en las cuales los factores medio-ambientales y socio-económicos predisponen a una mayor sensibilidad para desarrollar enfermedades asociadas a la resistencia a la insulina como DM2, ECV e hígado graso no alcohólico ^89^^,^^90^ a través de mecanismos adaptativos epigenéticos relacionados con la síntesis de substancias involucradas en la regulación de la ingesta alimentaria, de la sensibilidad a la insulina y de la inflamación sistémica ^91^-^95^. 

## Conclusiones y recomendaciones 

1. La prevalencia de Prediabetes en Colombia es alta y a pesar estar reconocida y categorizada en las principales Guías Médicas de la especialidad nacionales e internacionales, y de estar incluida en la Clasificación Internacional de Enfermedades (CIE-10) vigente en Colombia, el conocimiento que de ella tiene el equipo de salud y la comunidad es limitada, por lo que se recomienda que las Facultades de Ciencias de la Salud y las Asociaciones Científicas relacionadas con la educación médica continuada implementen acciones destinadas a mejorar los conocimientos del equipo de salud y de la comunidad en general sobre el significado que tiene el identificar, diagnosticar y tratar al paciente con prediabetes. El grupo de expertos recomienda también que en los programas educativos que deben implementarse se insista en la necesidad de un enfoque global del riesgo incluyendo la importancia de conocer y evaluar otros factores de riesgo como antecedentes familiares de DM2, sobrepeso y obesidad, dislipidemia e hipertensión. El Ministerio de Salud debe hacer evidente el código de la prediabetes y divulgarlo para que la enfermedad pueda aparecer como un diagnóstico específico en las historias clínicas de los pacientes. 

2. Los dos estudios realizados en Colombia demuestran la utilidad de la encuesta FINDRIS para identificar sujetos en riesgo de prediabetes y DM2 por lo que se recomienda que la aplicación del FINDRIS debe ser una política gubernamental a ser aplicada por todas las instituciones promotoras y prestadoras de salud en el país. Su diligenciamiento debe hacerse a todos los pacientes adultos antes de la consulta por parte de algún miembro del equipo de salud no médico. 

La aplicación de FINDRIS evitará realizar pruebas de laboratorio innecesarias y con ello un ahorro importante en el gasto en salud por lo que es fundamental implementar una mayor difusión de esta herramienta como una obligación de ser realizada a nivel de Atención Primaria.

Para tomar la decisión sobre cuál formulario utilizar si el FINDRIS completo validado en Colombia o la versión acortada es necesario definir los objetivos de la evaluación: en términos de programas amplios de salud pública, posiblemente el utilizar el formulario corto ayude a ahorrar tiempo, sin embargo desde el punto de vista clínico el utilizar el cuestionario acortado disminuye la posibilidad de hacer comparaciones con otras mediciones realizadas con el FINDRIS original, por lo que el Consenso recomienda utilizar cualquiera de las dos versiones para evaluar el riesgo de los pacientes y se insiste en que la aplicación de FINDRISC debe ser una política institucional que debe realizarse inclusive cuando el paciente llegue a la consulta con el resultado de la glucemia en ayunas

3. Existe un amplio cuerpo de evidencias que demuestran que la Prediabetes progresa hacia DM2 a una tasa anual de al menos el 10% y además que independientemente de la progresión a DM2 la Prediabetes es un factor de riesgo para desenlaces cardiovasculares por lo que el Comité recomienda que una vez detectada y diagnosticada, se inicie el manejo inmediato a través de cambios en el estilo de vida y realizar una nueva valoración a los 3 y 6 meses. Si el paciente no responde con una pérdida de peso de al menos el 5% y si no se normalizan los valores de HbA1C, iniciar el manejo farmacológico con una dosis de 500 mg/día de metformina, escalando hasta 1,500-1,700 mg/día, según tolerancia.
